# Sevoflurane exposure causes neuronal apoptosis and cognitive dysfunction by inducing ER stress *via* activation of the inositol 1, 4, 5-trisphosphate receptor

**DOI:** 10.3389/fnagi.2022.990679

**Published:** 2022-10-20

**Authors:** Qi Zhang, Yanan Li, Xupeng Wang, Chunping Yin, Qi Zhou, Junfei Guo, Juan Zhao, Xiaohui Xian, Zhiyong Hou, Qiujun Wang

**Affiliations:** ^1^Department of Anesthesiology, Children’s Hospital of Hebei Province Affiliated to Hebei Medical University, Shijiazhuang, China; ^2^Department of Anesthesiology, The Third Hospital of Hebei Medical University, Shijiazhuang, China; ^3^Department of Orthopaedics, The Third Hospital of Hebei Medical University, Shijiazhuang, China; ^4^Experimental Centre for Teaching, Hebei Medical University, Shijiazhuang, China; ^5^Department of Pathophysiology, Hebei Medical University, Shijiazhuang, China

**Keywords:** sevoflurane, cognitive dysfunction, IP3R, neuroapoptosis, calcium

## Abstract

The role of the inositol 1, 4, 5-trisphosphate receptor (IP3R) in hippocampal neuronal apoptosis and cognitive dysfunction induced by sevoflurane is currently unclear. Therefore, in this study, we investigated the role of the IP3R in endoplasmic reticulum (ER) stress and hippocampal neuronal apoptosis induced by sevoflurane in aged rats and isolated hippocampal neurons using both *in vivo* and *in vitro* experiments, including bioinformatics, functional enrichment analysis, gene set enrichment analysis, hematoxylin, and eosin staining, TUNEL assay, flow cytometry, western blot analysis and transmission electron microscopy. Furthermore, behavioral assessment was performed with the Morris water maze test. We identified 232 differentially expressed genes induced by sevoflurane exposure, including 126 upregulated genes and 106 downregulated genes. Sevoflurane exposure caused cognitive impairment and neuronal injury, and increased p-IP3R levels and ER stress. An IP3R inhibitor, 2-APB, suppressed these changes, while an IP3R agonist, FK-506, aggravated these changes. Together, these findings suggest that sevoflurane exposure causes marked cognitive dysfunction in aged rats and neuronal injury in isolated hippocampal neurons by activating the IP3R and inducing cytoplasmic calcium overload, thereby resulting in ER stress and hippocampal neuronal apoptosis.

**GRAPHICAL ABSTRACT:**
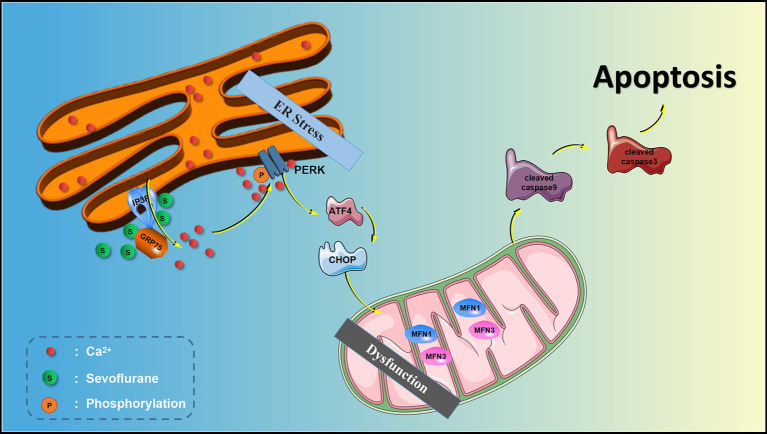

## Introduction

Postoperative cognitive dysfunction (POCD) refers to the postoperative impairment in cognition, learning, memory, orientation, and psychomotor behavior in patients without preoperative mental disorder after anesthesia and surgery (Gold and Forryan, 2018). The incidence of POCD 7 days after non-cardiac surgery can be as high as 9.1–17% (Evered et al., 2011; Krenk et al., 2014) and it can reduce the quality of life of patients and even increase mortality (Silbert et al., 2014). Accumulating evidence indicates that POCD is associated with two main risk factors: anesthesia and aging and sevoflurane inhalation could induce neurotoxicity and neurocognitive decline in elderly patients with POCD (Miller et al., 2018; Wang et al., 2018). POCD, however, still lacks a clear understanding of its underlying mechanisms (Chen et al., 2020; Ge et al., 2021; Wang et al., 2021). Therefore, a better understanding of the molecular mechanisms of POCD caused by sevoflurane is urgently needed.

Although the mechanisms underlying sevoflurane neurotoxicity are unclear, numerous studies have shown that neuroinflammation, calcium imbalance, neuronal apoptosis, and an increase in oxidative stress are closely related to sevoflurane-induced cognitive impairment (Ma et al., 2016; Chen et al., 2020; Wang et al., 2021). As a major intracellular messenger, calcium is involved in regulating the physiological activities of many cells and tissues, including muscle contraction, metabolism and cell division. In the resting physiological state, calcium ions are maintained at relatively high concentrations outside the cell and low concentrations inside the cell through homeostatic flux (Kumar, 2020; Sahu and Turner, 2021). When cells are stimulated, this calcium homeostasis is broken, and cytoplasmic calcium ([Ca^2+^]c) levels increase instantaneously, inducing cell damage or death, such as apoptosis and necroptosis under certain circumstances (Cheng et al., 2022; Matuz-Mares et al., 2022). Qiu LL et al. (Qiu et al., 2020) showed dysregulation of BDNF/TrkB signaling mediated by NMDAR/Ca^2+^/calpain caused by anesthesia/surgery might contribute to POCD in aging mice. Our previous studies showed that sevoflurane causes learning and memory deficits in rats by inducing neuroapoptosis by increasing [Ca^2+^]c and calcium pathway proteins such as calcineurin and calpain. Furthermore, we showed that inhibition of calcium overload alleviates the cognitive impairment induced by sevoflurane (Liu et al., 2016; Wang et al., 2021).

The endoplasmic reticulum (ER) is extremely sensitive to alterations in the intracellular environment, and cytoplasmic Ca^2+^ overload and oxidative stress caused by external stimuli will lead to ER dysfunction and trigger ER stress processes (Hotamisligil and Davis, 2016; Di Conza and Ho, 2020). The ER stress response can be accompanied by the activation of downstream double-stranded RNA-activated protein kinase-like ER kinase (PERK) and inositol-requiring enzyme-1 (IRE1). The activation of PERK and IRE1 promotes cellular apoptosis. Sevoflurane exposure can induce ER stress and hippocampal neuroapoptosis and cause cognitive impairment in aged and neonatal rats (Liu et al., 2017; Shen et al., 2018). The research from Yang et al. (2008) also suggested Inhalational anesthetics may induce cell damage by causing abnormal calcium release from the ER *via* excessive activation of IP3 receptors. Isoflurane has greater potency than sevoflurane or desflurane to cause calcium release from the ER and to induce cell damage.

The inositol 1, 4, 5-trisphosphate receptor (IP3R) is one of two calcium channels located on the ER membrane that play an important physiological role in normal cells. IP3R channel activity is regulated by redox status and by phosphorylation by various kinases, such as cAMP-dependent protein kinase (PKA), cGMP-dependent protein kinase (PKG), Ca^2+^/calmodulin-dependent protein kinase II (CaMKII) and different tyrosine kinases (Berridge, 2016). However, abnormal calcium release from the ER through overactivation of IP3R on the ER membrane may lead to abnormally elevated [Ca^2+^]c, mitochondrial calcium overload, and ER calcium depletion, all of which may lead to cell death (Egorova and Bezprozvanny, 2018; Mangla et al., 2020). We previously showed in isolated hippocampal neurons that the inhaled anesthetics sevoflurane and isoflurane can activate IP3R, increase hippocampal [Ca^2+^]c, and induce neuroapoptosis (Liu et al., 2016). Similarly, some studies have shown that activation of IP3R leads to calcium homeostatic imbalance, which can cause ER stress and induce apoptosis (Li et al., 2009; Yang et al., 2021). However, whether ER stress is involved in IP3R-activation-mediated cytoplasmic calcium disorder and neuroapoptosis induced by sevoflurane remains unknown.

The current evidence suggests that there is an intimate connection between ER stress and perturbed calcium homeostasis caused by IP3R activation in the neurotoxicity of sevoflurane in aged rats. Thus, we hypothesize that sevoflurane first activates IP3 receptors, causing calcium overload in the cytoplasm of hippocampal neurons, and then induces ER stress, causing hippocampal apoptosis and neurotoxicity. In this study, we test this hypothesis using bioinformatics analysis, behavioral experiments and molecular biology experiments, with the aim of clarifying the mechanisms underlying the clinical neurotoxicity of sevoflurane.

## Materials and methods

### Bioinformatics analysis

From the GENE EXPRESSION OMNIBUS (GEO) database^1^, we downloaded the postoperative cognitive impairment-related gene expression dataset (GSE95426). We used the R language limma package for quantile RNA-seq data standardization and to analyze differences in gene expression (|logFC| <2, *value of p* <1). The ggplot2 software package was used to process the GSE95426 dataset to generate a volcano map of differentially expressed genes (DEGs) in R software, and the R software package pheatmap was used to draw the cluster analysis heatmap of the DEGs.

### Functional enrichment analysis

The gene ontology (GO) and Kyoto Encyclopedia of Genes and Genomes (KEGG) analyses of the DEGs was conducted for the dataset GSE95426. Using the DAVID online database tools^2^, the level of biological processes for DEGs were analyzed by integrating the GO term and network to create the DEGs for a biological process. Ggplot2 and GOplot packages were used to map the GO pathway and enrichment analysis diagram of the KEGG pathway of DEGs in the R linguistic environment.

### Gene set enrichment analysis

GSEA^3^ was used for enrichment analysis of all genes, and the GSEA pathway was mapped.

### Animals and model preparation

**Figure1:**
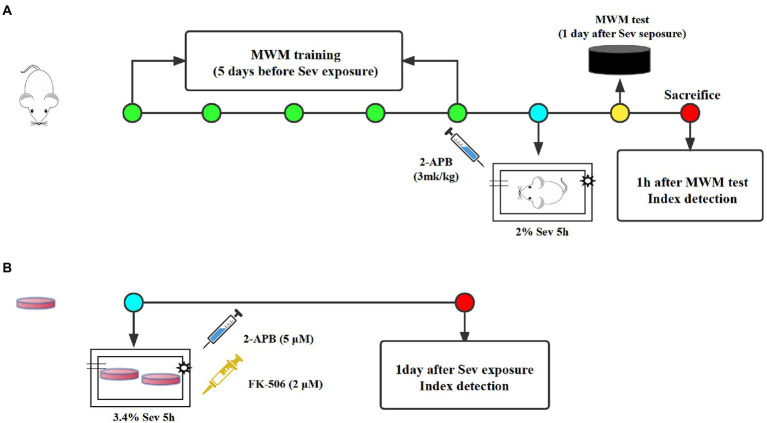
The experimental schematic diagram. **(A)** Rats received MWM training once a day for 5 days and were exposed to 3% sevoflurane for 2 h at 1 day after the last MWM training. At 1 day after sevoflurane exposure, MWM tests was established to evaluate cognitive function of aged rats, then 1 h after MWM tests, rats were sacrificed for index detection. **(B)** All dishes of hippocampal neurons were exposed to 3.4% sevoflurane for 5 h. At 1 day after sevoflurane exposure, all index were detected. MWM, Morris water maze, Sev, sevflurane.

Seventy-five healthy male Sprague–Dawley rats, 18 months of age, weighing 550–650 g, were purchased from the Experimental Animal Center of Hebei Medical University (License Number:SCXK2018-004). All animals were placed in a room maintained at a constant temperature and allowed free access to water and food. After a week of adaptive feeding, all rats were randomly divided into the following three groups (*n* = 25 each): Control, Sev, and Sev + 2-APB. Rats in the Sev and Sev + 2-APB groups were placed in an acrylic anesthetizing chamber with two interfaces; one was connected to a sevoflurane vaporizer (Drager, Germany) and the other was connected to a multi-gas monitor (Datex-Ohmeda, United States). The rats were exposed to 2% sevoflurane (Maruishi Pharmaceutical Co., Ltd. Japan) delivered in humidified 30% O_2_ carrier gas for 5 h according to our previous study (Yin et al., 2022). Rats in the Control group were exposed to humidified 30% O_2_ balanced by N_2_ in an acrylic anesthetizing chamber without sevoflurane. 2-APB (Cat# D9754; Sigma-Aldrich, United States), a IP3 receptor antagonist, were dissolved in 10% DMSO. Before anesthesia, 3 mg/kg 2-APB was injected intraperitoneally in Sev + 2-APB group, and the same amount of 10% DMSO was injected into the other two groups according to the previous research (Vaidya et al., 2022; Figure 1). The protocols were approved by the Animal Review Board of Hebei Medical University (Ethical code: 2017–026-1).

### Cell culture and processing

Primary rat hippocampal neurons were obtained from fetal rats for *in vitro* experiments, according to the method described by Patrich et al. (2016). Pregnant rats (16–18 days of gestation) were purchased from the Experimental Animal Center of Hebei Medical University (License Number: SCXK2018-2-003), and the fetal rats were taken by laparotomy immediately after deep anesthesia. The fetuses were placed in precooled equilibrium solution, decapitated, and the brain tissue was removed and placed in an ice-cold petri dish containing DMEM/F12. The hippocampal tissue was separated under an anatomical microscope, cut into 1 × 1 × 1 mm pieces, placed in a centrifuge tube, topped with 0.25% trypsin (HyClone, United States) of equal volume, and placed in a CO_2_ incubator at a constant temperature of 37°C. The centrifuge tube was shaken once every 3 min. After digestion for 10 min, an equal volume of DMEM containing 20% fetal bovine serum (Gibco, United States) was added to the centrifuge tube to terminate the trypsin reaction. After centrifugation, the supernatant was removed, the pellet was resuspended and filtered to collect the cells, and the morphology of neurons was observed under an inverted microscope and analyzed with the EVOS imaging system (EVOS M7000, Thermo Fisher Scientific, United States). If the neuronal cell bodies were plump and distinct, and the processes formed a dense network, the cells were included in the study. Hippocampal neurons were plated onto culture dishes pre-coated with 5 μg/mL poly-L-lysine at a density of 5 × 10^5^/mL, 2,000 μl/well. Then, 24 h later, the medium was replaced with medium for hippocampal neurons [Neurobasal +3% B27], and half of the medium was replaced twice a week. On day 7 of culture, histochemistry for the microtubule-associated protein MAP2 and DAPI was used to identify neurons. The neurons had plump cell bodies and processes that formed a dense network, with a purity of >95%, and therefore were used in subsequent experiments. All cultures of hippocampal neurons were divided into the following four groups: Control, Sev, Sev + 2APB, and Sev + FK-506. Each group of dishes was placed in an anesthesia induction chamber maintained at 37°C (RWD Life Science Co., Ltd., Shenzhen, China) containing fresh gas (21% O_2_, 5% CO_2_ and 69% N_2_) (Control group) or with the addition of 3.4% sevoflurane for 5 h (Sev, Sev + 2APB, Sev + FK-506 group) according to the previous study (Xu et al., 2018). A steady level of 3.4% sevoflurane was maintained at a gas flow rate of 1 l/min, measured with a multi-gas monitor (Datex-Ohmeda). Then, 1 h before sevoflurane exposure, 2-APB (cell permeable IP3R inhibitor, 5 μM) and FK-506 (activator of IP3R, 2 μM) were added into the culture medium in the Sev + 2APB and Sev + FK-506 groups, respectively, according to the method described by Fujii (Fujii et al., 2016; Figure 1). 2-APB and FK-506 were obtained from Sigma-Aldrich.

### Morris water maze test

The memory and learning ability were evaluated by MWM test. For the MWM experiment, we used an indoor stainless steel drum (1,500 mm in diameter and 500 mm in height), with a black inner surface, filled with warm water (24–26°C). The water in the pool was made opaque after adding nontoxic black ink, which was then divided into 4 quadrants: I, II III, and IV, and in the middle part of quadrant IV,a circular platform of 12 cm in diameter and 30 cm in height was positioned at 2 cm below the surface of the water. In addition, the reference substance and the room lights were maintained around the pool. The spatial acquisition experiment was conducted on all animals for 5 consecutive days before sevoflurane exposure. During training period, rats were dropped into the water at four different starting positions facing the wall, then guided to board the platform and remain on it for 15 s if they failed within 120 s. Cleaning the pool every day after training eliminated the smell prompt. The rats trained for four sessions per day. According to our previous study (Yin et al., 2022), 24 h after sevoflurane exposure, the learning and memory ability was tested using the positional navigation experiment. The time from entering the water to finding the hidden platform in the contralateral quadrant was recorded. Then, the platform was removed, and spatial memory ability was evaluated positioning navigation experiment and space exploration experiment. The number of crossings of the platform, the time spent in the target quadrant and swimming speed were recorded.

### Hematoxylin and eosin staining

At 1 h after the MWM test, five mice from each group were sacrificed, and brain tissues were collected and rinsed with phosphate buffer solution. The tissues were then immersed in 4% paraformaldehyde for 24 h. Subsequently, the samples were dehydrated and embedded in paraffin wax. The paraffin-embedded coronal brain sections (5 μM) containing the hippocampus were cut and HE-stained. The pathological changes in the hippocampus were examined by optical microscopy.

### TUNEL assay

At 1 h after the MWM test, five aged mice from each group were sacrificed, the brain tissues were removed and embedded in OCT medium, and then placed in the −80°C freezer. Frozen sections at a thickness of 10 μM were fixed with 4% paraformaldehyde for 30 min, rinsed with phosphate-buffered saline (PBS), fixed and treated with 3% BSA + 0.3% Triton X-100 for 1 h, and then TUNEL-stained. Briefly, after treatment with protease solution, the sections were incubated with equilibration buffer for 10 min, followed by incubation with the terminal DNA transferase reaction mixture containing fluorescent-labeled substrate at 37°C for 1 h in the dark. Finally, anti-fade solution containing DAPI was added. The number of apoptotic cells was counted with Image J software under the fluorescence microscope.

### Measurement of hippocampal [Ca^2+^]_c_ and apoptosis rate

The hippocampus tissues from each group (*n* = 5) were harvested 1 h after the MWM test and prepared into cell suspensions, as previously described (Liu et al., 2016; Zhang et al., 2018). A 200-mesh nylon sieve was employed for filtration of the hippocampal tissues (1 g), followed by centrifugation at 1310 × g for 5 min at 4°C and removal of the supernatant (n = 5). The cells were then washed twice with PBS and centrifuged to prepare single cell suspensions at a concentration of 1 × 105/mL. Subsequently, the single cell suspension was incubated with 5 μmol/l Fura-3 AM (Solarbio, IF0150) at 37°C for 30 min. After washing twice, cells were re-suspended in PBS at 37°C for 15 min. Flow cytometry was performed to measure fluorescence intensity, with an excitation wavelength of 480 nm and an emission wavelength of 525 nm. The fluorescence intensity reflects the concentration of intracellular calcium.

The cell suspension was prepared by the same method as above, resuspending the cells in 500 μl of 1 × binding buffer after centrifugation. Subsequently, added 5 μL of 20 μg/mL Annexin V-FITC and 10 μL of 50 mg/mL PI (Invitrogen, 282,932–000), incubated at room temperature for 10 min in the dark. The cells were washed and analyzed by FACS Calibur (Becton, Dickinson Company, United States). The percentages of cells in each quadrant were analyzed by FACS Calibur (FC500; Beckman Coulter Inc.) using ModFit software (EXPO32 ADC v1.2; Beckman Coulter Inc.). The apoptosis rate was detected by calculating the (Annexin V-FITC)+/PI+ and (Annexin V-FITC)-/PI+ cells numbers.

### Ultrastructure of hippocampal neurons

Hippocampal tissues from the CA1 region from each group (*n* = 5) were collected (approximately 1 mm × 1 mm × 3 mm), fixed with 4% glutaraldehyde and 1% osmium tetroxide, dehydrated through a series of ethanol solutions, and then embedded in epoxy resin and double stained with uranyl acetate and lead citrate. The ultrastructure of hippocampal neurons was observed under a transmission electron microscope (H-7500, Hitachi, Japan).

### Western blot

Total protein extraction was performed using the Whole Cell and Tissue Protein Extraction Kit (Thermo Fisher Scientific, United States) according to the manufacturer’s protocol (*n* = 5). Protein concentrations were measured using the BCA Protein Assay Kit (A53225, Thermo Fisher Scientific). The proteins were separated by 10% SDS-PAGE and transferred onto a PVDF membrane. After blocking with 5% skim milk powder for 2 h, the membrane was incubated overnight with primary antibody [p-IP3R (ab111615), total-IP3R (ab264281), MFN1 (ab221661), MFN2 (ab260861), GRP75 (ab171089), p-PERK (ab192591), ATF4 (ab184909), CHOP (ab11419), cleaved caspase-3 (ab32042), cleaved caspase-9 (ab184786), Bax (ab32503) and Bcl-2 (ab32124) were purchased from Abcam and diluted to 1: 1,000 before use). In this study, β-actin (ab8226, 1: 2000) served as the internal reference protein. After washing, the membrane was incubated with secondary antibody for 2 h. The ultra-sensitive chemiluminescent liquid-based FujiFilm LAS 4000 imaging analyzer (FujiFilm, Tokyo, Japan) was used for visualization, and Image J (NIH, Bethesda, MD, United States) was used to analyze the relative intensities of individual bands.

### Immunohistochemistry

The brain was prepared as described in Method 2.3, sliced into 15 mm sections on a cryostat, blocked in PBS containing 1% goat serum and 0.1% Triton-X 100, and incubated at 4°C overnight with anti-p-IP3R (ab111615). After washing, a streptavidin horseradish peroxidase (HRP) complex (1: 1000; Dako) was applied for 1 h. Color development was performed with a diaminobenzidine peroxidase substrate kit (Vector Labs, Burlingame, CA, United States). Sections were counterstained with hematoxylin or eosin. The sections were observed and photographed under an inverted light microscope, and the positive cell rate was calculated by Image Pro Plus 6.0 software (Media Cybernetics, Inc., Rockville, MD, United States).

### Statistical analysis

SPSS 21.0 software was used for statistical analysis. The data meet normal distribution were expressed as mean ± standard deviation, and differences between groups were assessed by one-way analysis of variance (ANOVA), followed by the Turkey multiple comparison analysis. *p* < 0.05 indicated that the difference was statistically significant.

## Results

### Screening of differentially expressed genes

**Figure 2:**
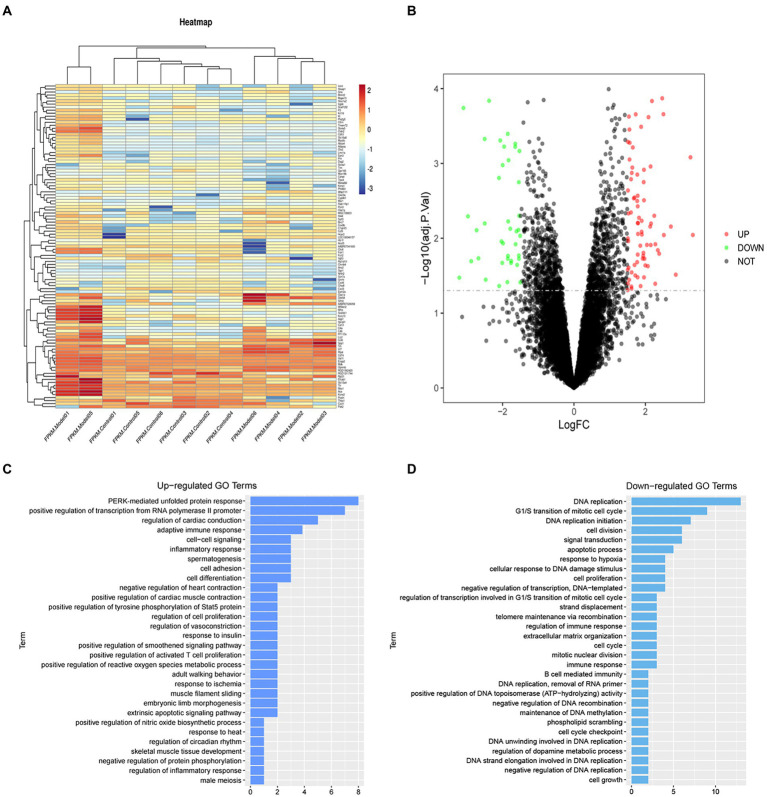
**(A)** Heatmap of differential gene cluster analysis. **(B)** Differential gene volcano map. **(C)** GO enrichment analysis upregulated pathways. **(D)** GO enrichment analysis downregulated pathways.

To analyze gene expression differences of sevoflurane’s neurotoxicity, the dataset GSE95426 was downloaded from the GEO database, and quantile standardization was performed on the data. With *value of p* <0.05 and |logFC| <2 as criteria for screening GSE95426, a total of 232 DEGs were retrieved, among which 126 were upregulated and 106 were downregulated. The ggplot2 software package was used to construct the visual group DEG volcano map of GSE95426 in R software (Figure 2), and the R software package pheatmap was used to draw the cluster analysis heatmap of the DEGs (Figure 2).

### Results of bioinformatics analysis

**Figure 3:**
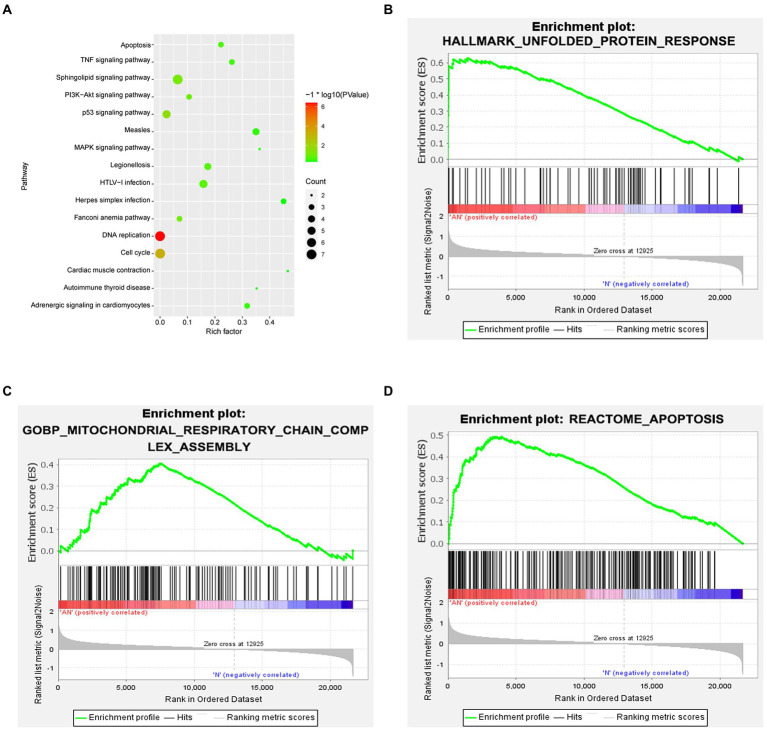
**(A)** KEGG enrichment analysis revealed that pathways such as Apoptosis and DNA replication were enriched. **(B–D)** GSEA gene enrichment analysis revealed that the unfolded protein response, mitochondrial respiratory chain complex assembly and apoptosis pathways were significantly enriched pathways.

Functional enrichment analysis of DEGs were performed by GO and KEGG enrichment analyses. Using DAVID online database tools (see text footnote 2), thelevelofbiological processes for DEGs were analyzed by integrating the GO term and network to identify the DEGs in a biological process. Upregulated and downregulated GO pathway maps of DEGs (Figure 2) were drawn by R language (Figure 2). GO pathway diagram revealed PERK-mediated unfolded protein response and positive regulation of transcription from RNA polymerase II promoter, regulation of cardiac conduction are the top 3 enriched GO terms among the upregulated DEGs, DNA replication, G1/S transition of mitotic cell cycle and DNA replication initiation are the top 3 enriched GO terms among the downregulated DEGs.. DEGs were used to analyze and map the KEGG pathways (Figure 3). From the KEGG pathway map, we found that pathways such as Apoptosis and DNA replication were enriched. In addition, through GSEA, we found that UNFOLDED_PROTEIN_RESPONSE (enrichment score: 0.63, *p*<0.001), MITOCHONDRIAL_ RESPIRATORY_ CHAIN_ COMPLEX_ASSEMBLY (enrichment score: 0.47, *p*<0.001) and APOPTOSIS (enrichmeand nt score: 0.58 *p* = 0.001) were significantly enriched pathways (Figure 3).

### Sevoflurane impairs spatial learning and memory abilities in aged rats

**Figure 4:**
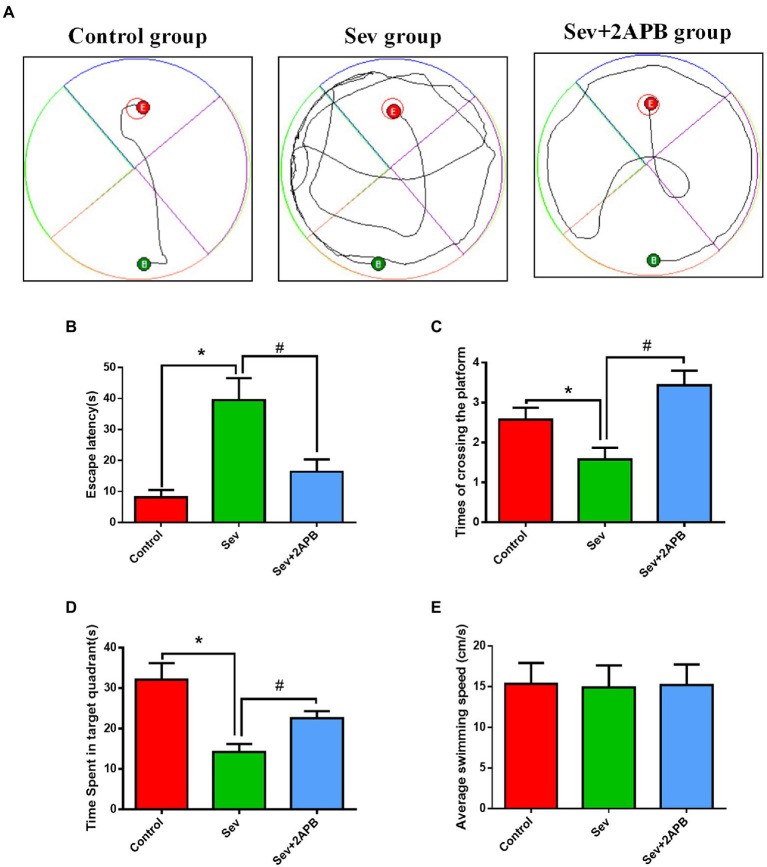
Sevoflurane impairs spatial learning and memory abilities in aged rats. (**A**) Swimming trajectory in the spatial exploration experiment. **(B)** Average escape latency. (**C**) Number of crossings of the platform area. (**D**) Time spent in the target quadrant. (**E**) Average swimming speed in the MWM test. Data are presented as mean ± SD (*n* = 25 in each group). Compared with Control group, ^*^p < 0.05; compared with Sev group, ^#^*p* < 0.05.

The Morris water maze test was carried out to evaluate the cognitive ability of aged rats after sevoflurane exposure. The trajectories of the animals, the number of crossings over the original platform location and the percent time spent in the target quadrant were evaluated in the spatial probe trial. Compared with the Control and Sev + 2-APB groups, the swimming trajectory of the Sev group was more complex, and the time to find the platform was increased (Figure 4A). The escape latency, number of platform crossings and time spent in the target quadrant were significantly decreased in the Sev group compared with the Control group. The escape latency, number of platform crossings and time spent in the target quadrant were increased in the Sev + 2-APB group compared with the Sev group (Figures 4B–D). However, there were no significant differences in swimming speed among the three groups (Figure 4E).

### Sevoflurane causes neuronal injury in aged rats

**Figure 5:**
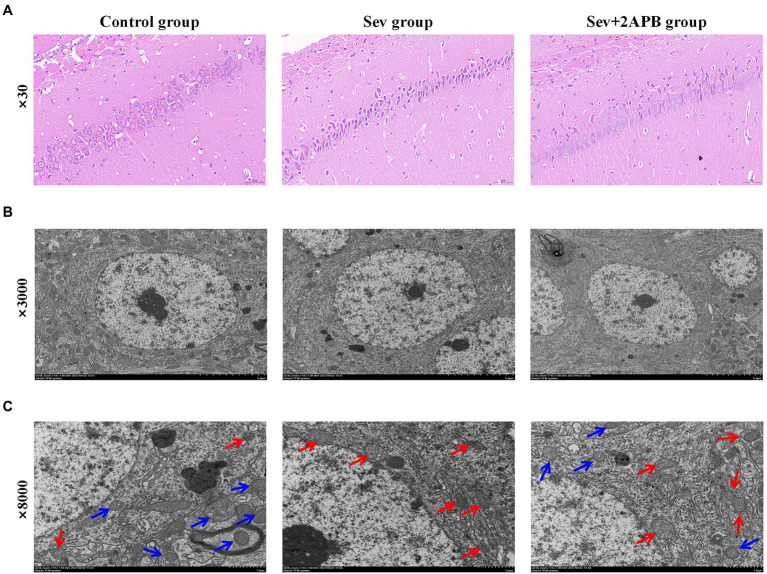
Sevoflurane promotes neuronal injury in aged rats. (A) Representative images of histopathological changes in the hippocampal CA1 region of aged rats (× 30). (**B)** Representative images of hippocampal neurons in the CA1 region under the transmission electron microscope (× 3,000). **(C)** Representative images of hippocampal neurons in the CA1 region under the transmission electron microscope (× 8,000). *n* = 5 in each group.

We used HE staining to investigate the Pathological changes of hippocampus after sevoflurane exposure. The results revealed that neurons in the hippocampal CA1 region were arranged in an orderly manner, with normal morphology, a distinct cell membrane, round nucleus and clear nucleolus in the Control group. Neurons in the hippocampal CA1 region were arranged in a disordered manner, appeared shrunken and degenerated, the nuclear boundary was unclear, the nucleolus was undetectable, and showed signs of interstitial edema in the Sev group. Atrophy and degeneration of neurons and interstitial edema in the hippocampal CA1 region in the Sev + 2-APB group were less pronounced compared with the Sev group (Figure 5A).

We used transmission electron microscopy to evaluate hippocampal ultrastructure in the different groups at magnifications of ×3,000 (Figure 5B) and ×8,000 (Figure 5C). The blue arrow shows that the mitochondrial ridge structure is complete, without fracture, indicating the viability of hippocampal neurons. The red arrow indicates that the mitochondrial ridge structure is broken or even absent, and that the mitochondria are pyknotic, indicating the apoptosis of hippocampal neurons. These results demonstrate that compared with the Control group, hippocampal apoptosis caused by mitochondrial damage was significantly increased in the Sev group, and that these pathological changes were ameliorated (less severe) in the Sev + 2-APB group.

### Sevoflurane promotes hippocampal neuroapoptosis in aged rats and isolated hippocampal neurons

**Figure 6:**
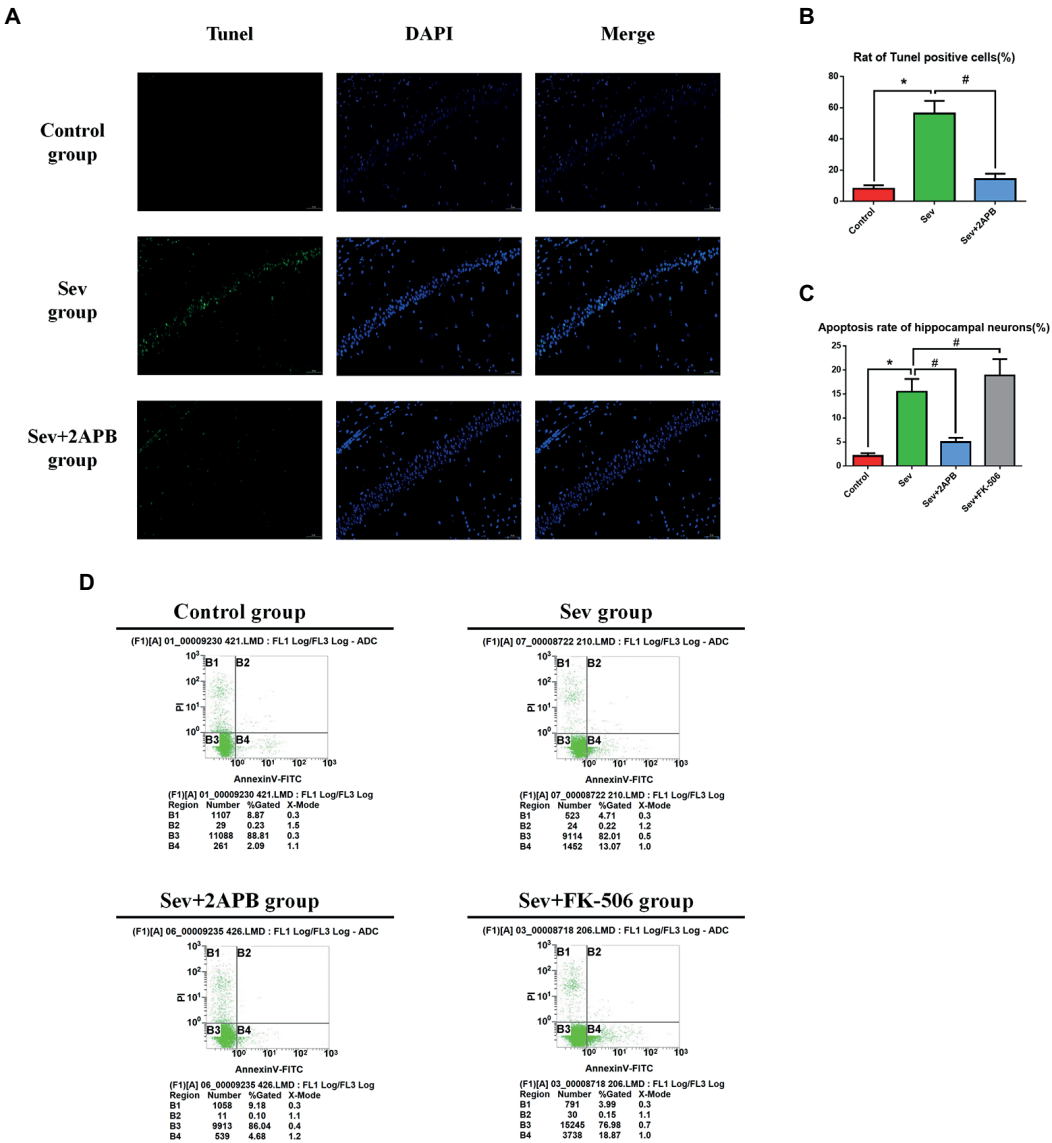
Sevoflurane promotes hippocampal neuroapoptosis in aged rats and isolated hippocampal neurons. **(A)** Representative images of TUNEL staining in the hippocampal CA1 region of aged rats (× 30) (green: Tunel positive cells; blue: DAPI, scale bar = 50 μM). **(B)** Numbers of TUNEL-positive cells. **(C)** Apoptosis rate of hippocampal neurons. **(D)** Representative flow cytometry of hippocampal apoptosis. Data are presented as mean ± SD (*n* = 5 in each group). Compared with Control group, ^*^p < 0.05; compared with Sev group, ^#^*p* < 0.05.

We used TUNEL staining to assess hippocampal neuroapoptosis in aged rats and flow cytometry (annexin V and PI double staining) to detect apoptosis in isolated hippocampal neurons. The *in vivo* experiments revealed that TUNEL-positive neurons were increased in the Sev group compared with the Control group (*p* < 0.01). In comparison, in the Sev + 2-APB group, the IP3R inhibitor 2-APB decreased the number of TUNEL-positive neurons (*p* < 0.01) (Figures 6A,B). The *in vitro* experiments showed that the apoptosis rate in hippocampal neurons was increased after sevoflurane exposure in the Sev group, compared with the Control group (*p* < 0.01). Compared with the Sev group, hippocampal neurons exhibited a notably reduced rate of apoptosis in the Sev + 2-APB group and an increased apoptosis rate in the Sev + FK-506 group after FK-506 (activator of IP3R) exposure (*p* < 0.01, Figures 6C,D).

### Sevoflurane elevates [Ca^2+^]_c_ in aged rats and isolated hippocampal neurons

**Figure 7:**
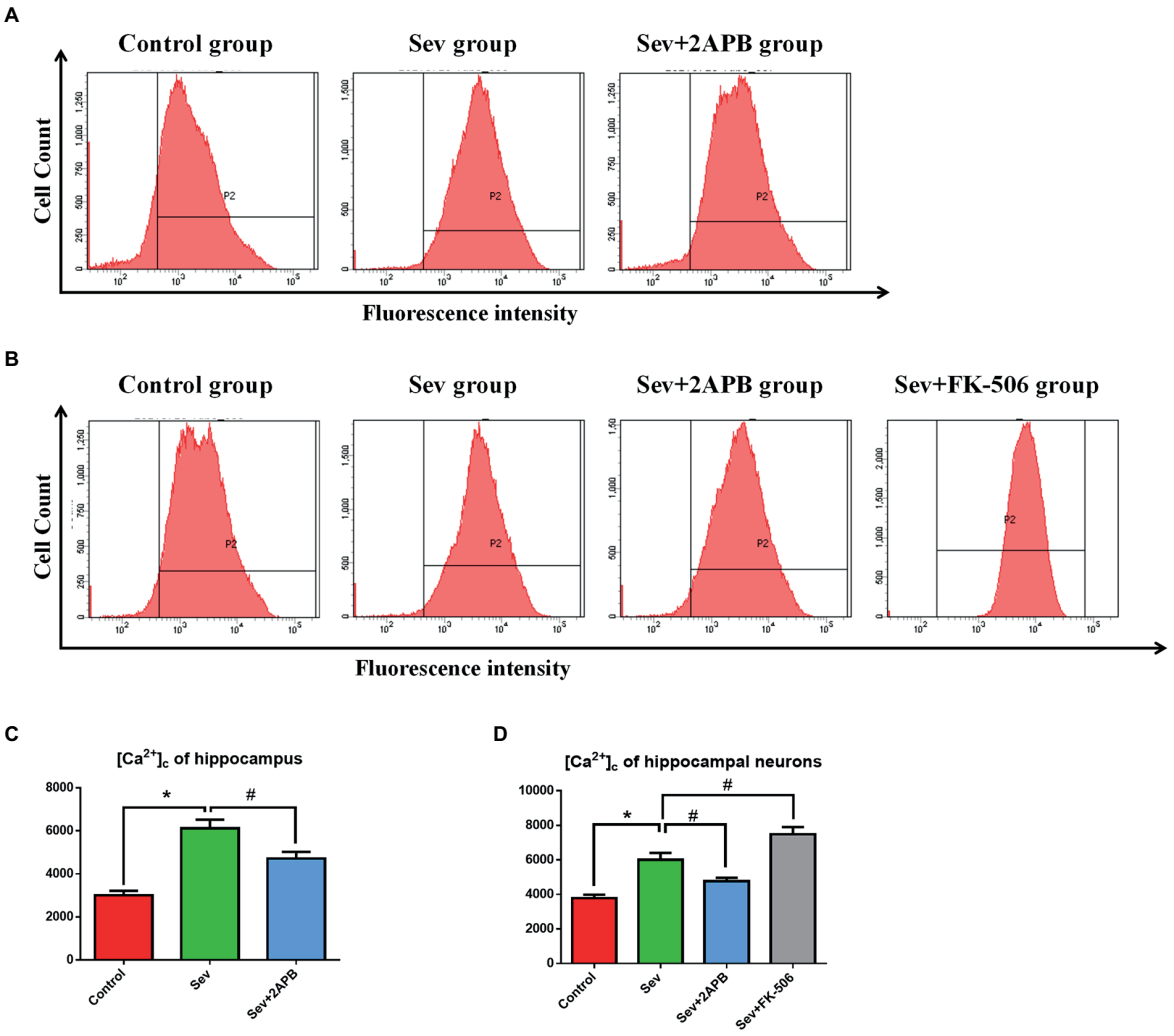
Sevoflurane elevates [Ca^2+^]_c_ in aged rats and isolated hippocampal neurons. **(A)** Intracellular [Ca^2+^]_c_ in hippocampal cells of aged rats. **(B)** Intracellular [Ca^2+^]_c_ in isolated hippocampal neurons. **(C)** Representative histograms of [Ca^2+^]_c_ by flow cytometry in the hippocampus of aged rats. **(D)** Representative histograms of [Ca^2+^]_c_ by flow cytometry of isolated hippocampal neurons. Data are presented as mean ± SD (*n* = 5 in each group). Compared with the Control group, ^*^p < 0.05; compared with the Sev group, ^#^*p* < 0.05.

To evaluate changes in intracellular calcium, we used the Ca^2+^ probe Fluo-3 AM and flow cytometry to measure [Ca^2+^]_c_. The *in vivo* experiments showed that [Ca^2+^]_c_ was significantly increased in the Sev group after sevoflurane exposure compared with the Control group (*p* < 0.01). In comparison, [Ca^2+^]_c_ was decreased in the Sev + 2-APB group (*p* < 0.01) (Figures 6A,C). The *in vitro* experiments showed that [Ca^2+^]_c_ was significantly increased after sevoflurane exposure in the Sev group, compared with the Control group (*p* < 0.01). Compared with the Sev group, the hippocampal neurons exhibited markedly reduced [Ca^2+^]_c_ in the Sev + 2-APB group and increased [Ca^2+^]_c_ in the Sev + FK-506 group (*p* < 0.01, Figures 7B,D).

### Sevoflurane increases the expression of p-IP3R and activates endoplasmic reticulum stress in aged rats

**Figure 8:**
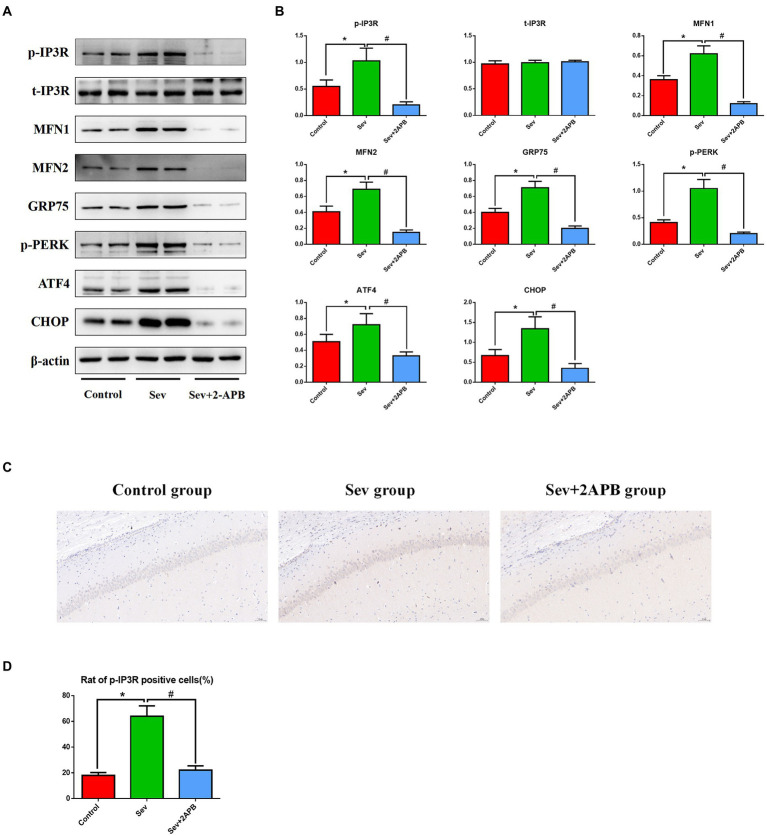
Sevoflurane upregulates p-IP3R and activates endoplasmic reticulum stress in aged rats. **(A)** Representative western blot of p-IP3R, t-IP3R, MFN1, MFN2, GRP75, p-PERK, ATF4 and CHOP. **(B)** Representative histogram of the relative expression of p-IP3R, t-IP3R, MFN1, MFN2, GRP75, p-PERK, ATF4, and CHOP. **(C)** Representative images of immunohistochemistry of p-IP3R in CA1 (scale bar = 50 μM). **(D)** Representative histogram of positive cell rate of p-IP3R. Data are presented as mean ± SD (*n* = 5 in each group). Compared with the Control group, ^*^p < 0.05; compared with the Sev group, ^#^*p* < 0.05.

To explore the potential mechanism of sevoflurane in reducing postoperative cognitive function in aged rats, we evaluated the expression of PERK-related ER stress markers in hippocampus by Western blot assay. The results revealed that sevoflurane exposure upregulated p-IP3R and PERK-related ER stress markers: MFN1, MFN2, GRP75, p-PERK, ATF4, and CHOP in the Sev group. In the Sev + 2-APB group, the IP3R inhibitor 2-APB suppressed the increase in these proteins induced by sevoflurane. There were no significant differences in total IP3R (t-IP3R) levels among the three groups (Figures 8A,B). To show activated IP3R in hippocampal neurons by sevflurane, we also performed immunohistochemistry of p-IP3R in CA1. The results showed the positive cell rate of p-IP3R was higher in group Sev than that in group C; moreover, the positive cell rate of p-IP3R was was lower in group Sev + 2APB than in group Sev (Figures 8C,D).

### Sevoflurane upregulates IP3R, increases ER stress and activates the caspase-related apoptotic pathway in isolated hippocampal neurons

**Figure 9:**
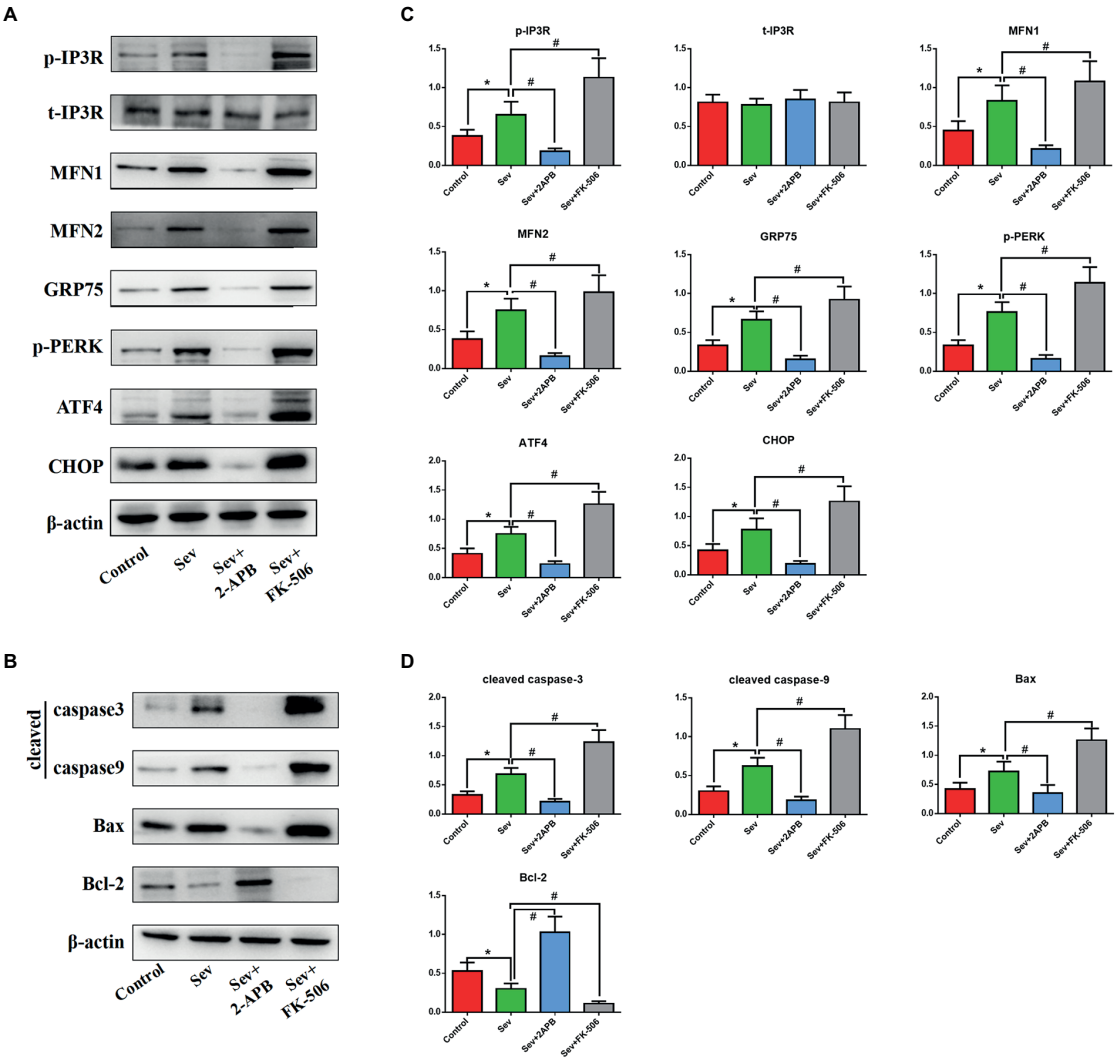
Sevoflurane upregulates IP3R and activates endoplasmic reticulum stress and the caspase-related apoptotic pathway in isolated hippocampal neurons. **(A)** Representative western blot of p-IP3R, t-IP3R, MFN1, MFN2, GRP75, p-PERK, ATF4, CHOP, cleaved caspase-9, cleaved caspase-3, Bcl-2, and Bax. **(B)** Representative histogram of the relative expression of p-IP3R, t-IP3R, MFN1, MFN2, GRP75, p-PERK, ATF4, CHOP, cleaved caspase-9, cleaved caspase-3, Bcl-2 and Bax. Data are presented as mean ± SD (*n* = 5 in each group). Compared with the Control group, ^*^p < 0.05; compared with the Sev group, ^#^*p* < 0.05.

To corroborate the findings in aged rats, we performed Western blot on isolated hippocampal neurons to evaluate the expression levels of the above proteins. Expression of signaling pathway proteins and apoptosis-related proteins was assessed by western blot analysis, with β-actin as a loading control. Representative blots and bar graphs from four independent experiments are shown. The results showed sevoflurane increased p-IP3R levels and upregulated ER stress-related proteins, including PERK-related ER stress markers: MFN1, MFN2, GRP75, p-PERK, ATF4 and CHOP. Furthermore, sevoflurane increased levels of cleaved caspase-9, cleaved caspase-3 and Bax, but downregulated the anti-apoptotic protein Bcl-2 in the Sev group. In comparison, 2-APB reduced p-IP3R levels and downregulated ER stress-related proteins and proapoptotic proteins, but upregulated anti-apoptotic proteins in the Sev + 2-APB group. In contrast, the IP3R agonist FK-506 upregulated p-IP3R and ER stress-related proteins and proapoptotic proteins, but decreased anti-apoptotic proteins in the Sev + FK-506 group. There were no significant differences in t-IP3R among the four groups (Figures 9A,B).

## Discussion

A large number of studies have shown that sevoflurane exposure is an important risk factor for POCD, and can result in increased mortality, delayed recovery, additional complications, longer hospital stays, and significantly higher medical costs (Ge et al., 2021; Wang et al., 2021). However, the mechanisms underlying sevoflurane-induced POCD are still unclear. The current findings provide new mechanistic insight into how sevoflurane exposure in aged rats induces cognitive impairment. Notably, the DEGs associated with cognitive dysfunction caused by sevoflurane were mainly concentrated in the PERK-mediated unfolded protein response and apoptosis pathways. The *in vivo* and *in vitro* experiments suggest that sevoflurane causes neuronal injury and cognitive dysfunction by activating IP3R and inducing ER stress, and by promoting apoptosis of hippocampal neurons through cytoplasmic calcium overload.

Medical bioinformatics is an interdisciplinary approach that uses computer science as a tool to store, retrieve, analyze, and interpret biological and medical data (Chattopadhyay et al., 2020). The rapid development of gene chip and high-throughput sequencing technologies has allowed researchers to quickly perform detailed analysis of the transcriptome and genome, which has promoted the rapid development and progress of the life sciences. In this study, we downloaded the rat sevoflurane expression-related dataset from the official GEO website, performed quantile standardization in R language, and analyzed the differential genes. The differential genes were analyzed by GO enrichment analysis, KEGG enrichment analysis and GSEA analysis. The analyses revealed that the ER stress and apoptosis pathways were the most markedly affected by sevoflurane exposure, laying the course for further *in vivo* and *in vitro* experiments.

Sevoflurane is the most commonly used halogen inhalation anesthetic in China. Sevoflurane anesthesia is closely related to cognitive decline, especially in elderly patients. Our previous study (Yin et al., 2022) showed that continuous exposure to 2% sevoflurane for 5 h significantly impairs cognitive function in aged rats. Therefore, using this animal model, we tested cognitive function with the MWM test 1 day after sevoflurane anesthesia, and we examined the morphology of hippocampal neurons by transmission electron microscopy and HE staining. The MWM is a common method for evaluating the spatial cognitive function of rodents. It is divided into a positional navigation experiment and a spatial exploration experiment. The position navigation experiment reflects the spatial learning ability of animals, while the spatial exploration experiment reflects spatial memory ability (Ravichandran et al., 2018). The shorter escape latency and the increase in the number of crossings of the original platform location indicate better learning and memory abilities. Our results revealed that the cognitive function of aged rats was significantly reduced and that hippocampal neurons were severely damaged after sevoflurane anesthesia, consistent with previous studies (Yin et al., 2022).

The ER, as the intracellular calcium store, plays an important role in maintaining intracellular calcium homeostasis. IP3R is an important calcium release channel located in the ER. The overactivation of IP3R can lead to the release of ER calcium into the cytoplasm, induce ER stress, and cause programmed cell death (such as apoptosis and necroptosis). A study by Yang found that sevoflurane may induce cell damage by causing abnormal calcium release from the ER *via* excessive activation of IP3 receptors (Yang et al., 2008), and IP3R knockout can prevent this damage. A study by Wang found that disruption of intracellular calcium balance by activation of IP3R results in hippocampal apoptosis, which underlies subsequent spatial memory impairment in mice (Wang et al., 2018). Previous studies found that 3.4% sevoflurane exposure for 5 h can significantly increase the apoptosis rate of isolated hippocampal neurons and cause nerve damage. Therefore, in this study, we chose 3.4% sevoflurane exposure for 5 h to explore the specific mechanism of sevoflurane neurotoxicity (Xu et al., 2018). In this study, we used FK-506 to activate IP3R, as in the study of Fujii (Fujii et al., 2016). FK-506 competes with IP3R for binding to FK506 binding protein (FKBP), and this process is irreversible. FK-506 allows IP3R to release FKBP, greatly increasing the activity of IP3R. Therefore, we used FK-506 as an agonist of IP3R (Mac Millan, 2013). Our current findings revealed that sevoflurane exposure activates IP3R and induces hippocampal apoptosis by disrupting intracellular calcium balance.

Apoptosis is a mechanism of programmed cell death caused by changes to the internal and external environments (Elmore, 2007). There are two apoptotic pathways in mammalian cells—the exogenous caspase 8 pathway, involving tumor necrosis factor receptor family members, and the endogenous caspase 9 pathway, involving cytochrome C (CytC)(D’Arcy, 2019). The process of apoptosis is regulated by a variety of genes, among which the Bcl-2 gene family is an important regulatory factor and plays various roles in apoptosis, depending on the family member. Bax is the most important apoptosis-inducing gene in the Bcl-2 family. It releases CytC into the cytoplasm through mitochondrial permeability transformation channel protein, forming the CytC-Apaf-pro-caspase-9 apoptotic complexes, which inhibit Bcl-2 and initiate the apoptotic cascade. Activation of the upstream effector caspase-9 can also activate downstream caspases, leading to apoptosis. Our previous study showed that sevoflurane exposure activates the caspase-dependent apoptotic pathway by inducing cytoplasmic calcium overload in hippocampal neurons (Liu et al., 2016). However, it was unclear whether ER stress is involved in this process.

ER stress is a process in which cells activate signaling pathways such as the unfolded protein response, ER overload response and the caspase-12-mediated apoptotic pathway in response to protein misfolding and aggregation in the ER lumen and calcium imbalance. ER stress not only induces the expression of ER molecular chaperones of glucose regulatory proteins (GRP78, GRP94) to exert protective effects, but also activates intrinsic apoptotic pathways. PERK is an important transmembrane sensor that senses and participates in the unfolded protein response in the mammalian ER. When the ER is stable, PERK and GRP75 are inactive. When the ER is stressed, PERK phosphorylates and catalyzes the phosphorylation of eIF2α through MFN1 and MFN2. Phosphorylated eIF2α selectively promotes the translation of ATF-4 and upregulates CHOP. CHOP increases the expression of the pro apoptotic protein Bax and cleaved-caspase-3/9 and downregulates the anti-apoptotic protein Bcl-2, thereby inducing apoptosis. Liu showed that sevoflurane exposure induces neuroapoptosis *via* the PERK-eIF2α-ATF4-CHOP axis of the ER stress signaling pathway (Liu et al., 2017). However, whether IP3R mediates cytoplasmic calcium remained unclear. In the current study, we found that after sevoflurane exposure, IP3R, [Ca^2+^]_c_ and neuronal apoptosis in the hippocampus were increased, and that the ER stress-related PERK pathway proteins and apoptotic proteins were increased, suggesting that sevoflurane exposure-induced neuroapoptosis is mediated by the activation of IP3R, which increases [Ca^2+^]_c_ and ER stress.

Although rigorous bioinformatics analysis and both *in vitro* and *in vivo* experiments were conducted in this study, there are some limitations. For example, no animal experiments with gene overexpression or knockout were conducted to further verify the findings. We plan on addressing this shortcoming in future studies. Additionally, the current study only evaluated the neurotoxic effects and related mechanisms of sevoflurane on aged male rats, and its effects on female and other age rats need to be further explored. Finally, we only explored PERK-induced ER stress, and the other two pathways (IRE1α-xbp1 and ATF6 signaling pathway) need to be further explored.

## Conclusion

Our data show that sevoflurane exposure causes cognitive impairment in aged rats and cellular damage in isolated hippocampal neurons. The mechanisms underlying the neurotoxicity of sevoflurane involve in activating the IP3R and inducing cytoplasmic calcium overload, thereby resulting in endoplasmic reticulum stress and hippocampal neuronal apoptosis.

## Data availability statement

The datasets presented in this study can be found in online repositories. The names of the repository/repositories and accession number(s) can be found in the article/Supplementary material.

## Ethics statement

The animal study was reviewed and approved by the Animal Review Board of Hebei Medical University (Ethical code: 2017-026-1).

## Author contributions

QZ, YL, CY, ZH, and QW: conception and design of study, bioinformatics analysis, and animal model construction. XW, QZ, JG, and JZ: acquisition of data, western blot, and cell culture experiment. XW, JG, and XX: analysis and/or interpretation of data, transmission electron microscope experiment. QZ and YL: drafting the manuscript. QZ, XW, and QW: revising the manuscript critically important intellectual content. All authors contributed to the article and approved the submitted version.

## Funding

This work was supported by the Natural Science Foundation of Hebei Province (H2022316001), Graduate Innovation Funding Project of Degree Office of Hebei Provincial Department of Education (CXZZBS2022092), and National Natural Science Foundation of China (81771134). The Hebei Provincial government funded the specialty capacity building and specialty leader training program.

## Conflict of interest

The authors declare that the research was conducted in the absence of any commercial or financial relationships that could be construed as a potential conflict of interest.

## Publisher’s note

All claims expressed in this article are solely those of the authors and do not necessarily represent those of their affiliated organizations, or those of the publisher, the editors and the reviewers. Any product that may be evaluated in this article, or claim that may be made by its manufacturer, is not guaranteed or endorsed by the publisher.

## Footnotes

^1^https://www.ncbi.nlm. nih.gov/gds/

^2^ https://david.ncifcrf.gov/

^3^ http://www.gsea-msigdb.org/
