# Supplementary figures and images for: Sevoflurane exposure causes neuronal apoptosis and cognitive dysfunction by inducing ER stress via activation of the inositol 1, 4, 5-trisphosphate receptor
Source: Front Aging Neurosci. 2022 Oct 20;14:990679. doi: 10.3389/fnagi.2022.990679 (PMC9631943; doi:10.3389/fnagi.2022.990679)

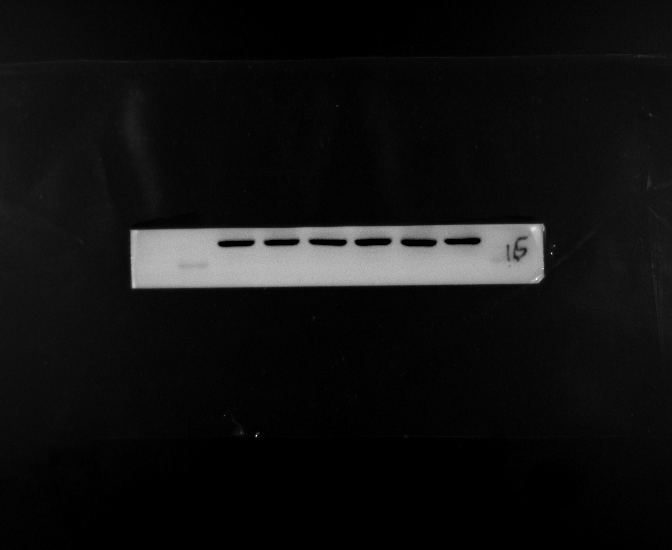

Supplement: Supplementary file 1 [file Data_Sheet_1.ZIP › 20210704-省三院-张琦-体内-全/actin(16)0616.tif]

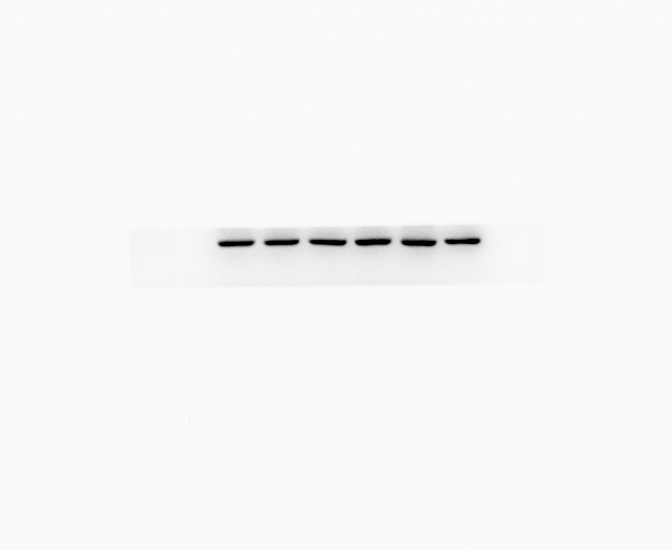

Supplement: Supplementary file 1 [file Data_Sheet_1.ZIP › 20210704-省三院-张琦-体内-全/actin(16)0616_chemi.tif]

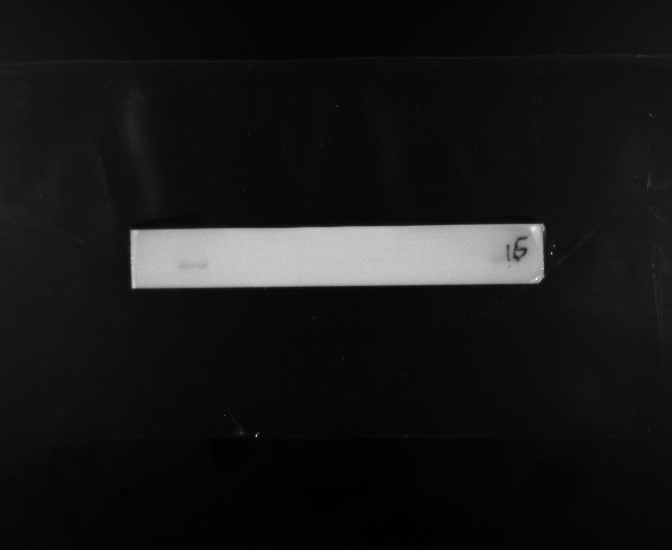

Supplement: Supplementary file 1 [file Data_Sheet_1.ZIP › 20210704-省三院-张琦-体内-全/actin(16)0616_marker.tif]

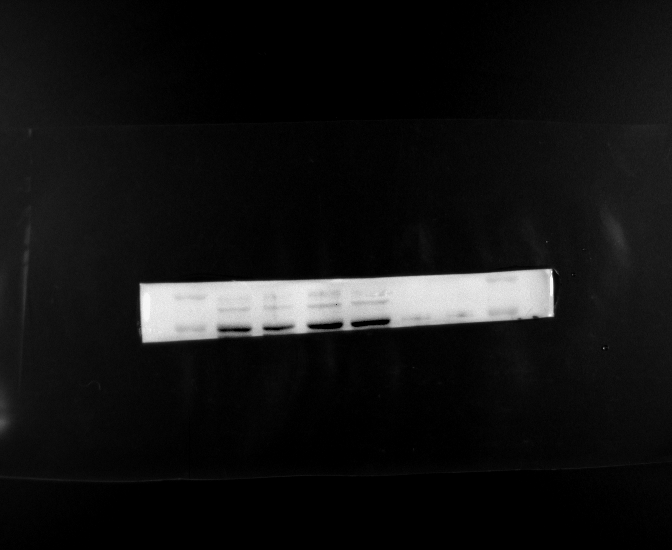

Supplement: Supplementary file 1 [file Data_Sheet_1.ZIP › 20210704-省三院-张琦-体内-全/ATF4(3-2)0707-1.tif]

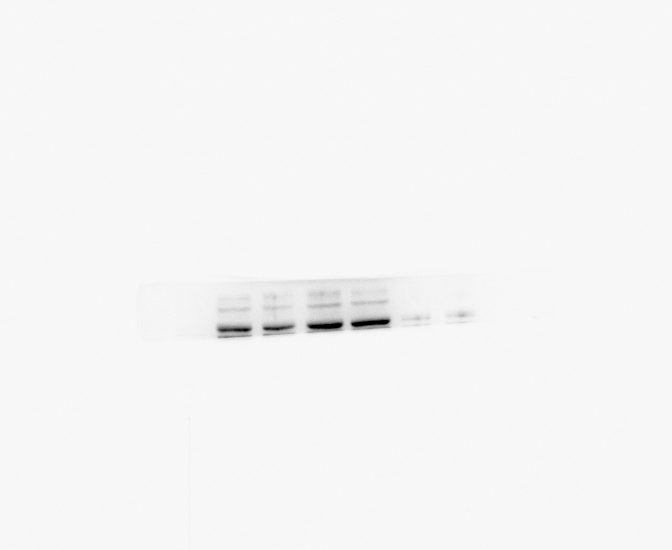

Supplement: Supplementary file 1 [file Data_Sheet_1.ZIP › 20210704-省三院-张琦-体内-全/ATF4(3-2)0707-1_chemi.tif]

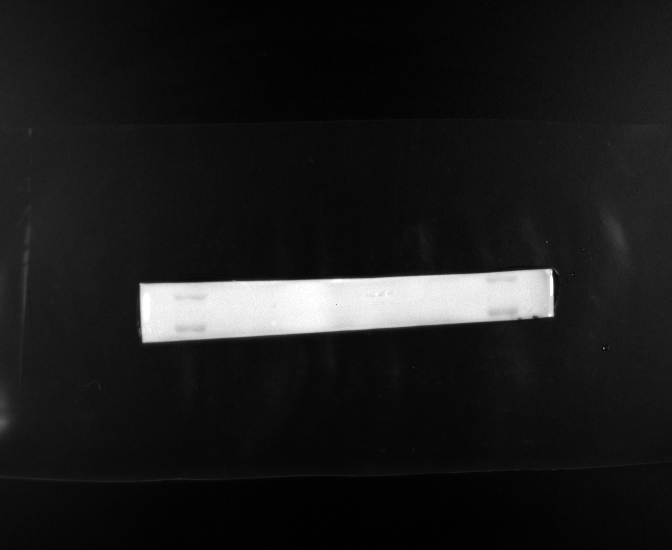

Supplement: Supplementary file 1 [file Data_Sheet_1.ZIP › 20210704-省三院-张琦-体内-全/ATF4(3-2)0707-1_marker.tif]

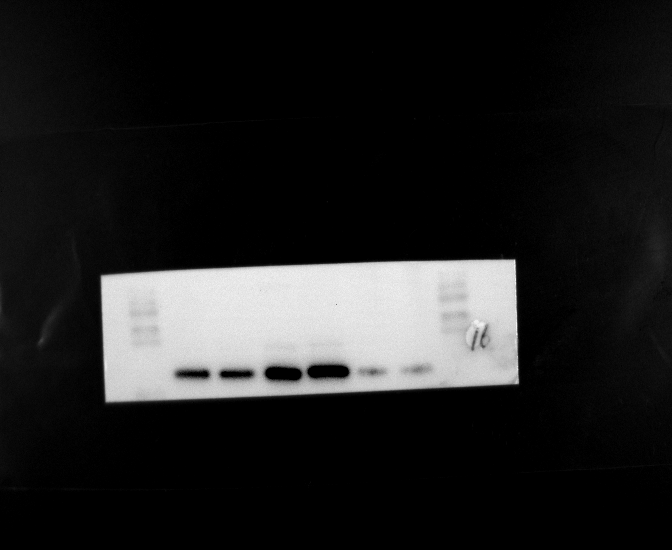

Supplement: Supplementary file 1 [file Data_Sheet_1.ZIP › 20210704-省三院-张琦-体内-全/CHOP(16)0705.tif]

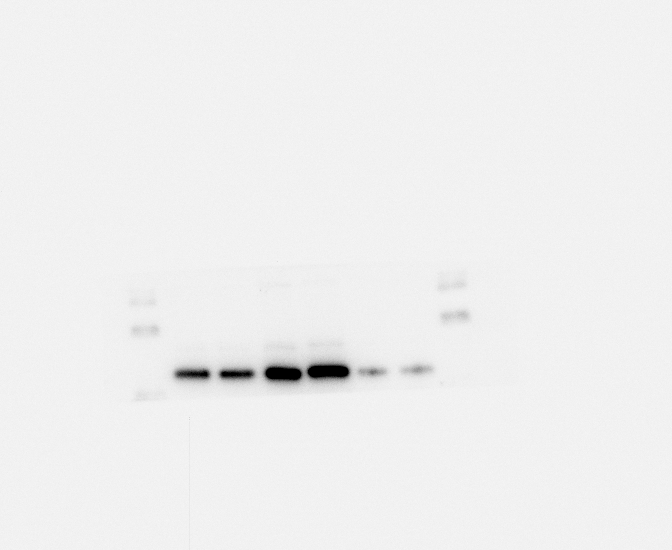

Supplement: Supplementary file 1 [file Data_Sheet_1.ZIP › 20210704-省三院-张琦-体内-全/CHOP(16)0705_chemi.tif]

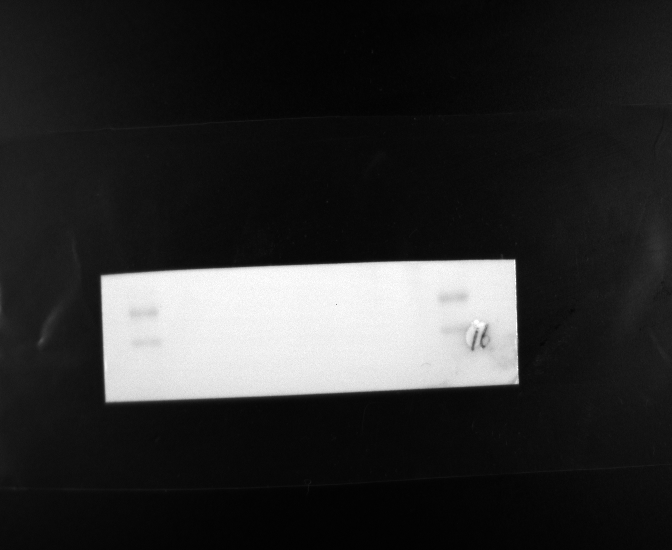

Supplement: Supplementary file 1 [file Data_Sheet_1.ZIP › 20210704-省三院-张琦-体内-全/CHOP(16)0705_marker.tif]

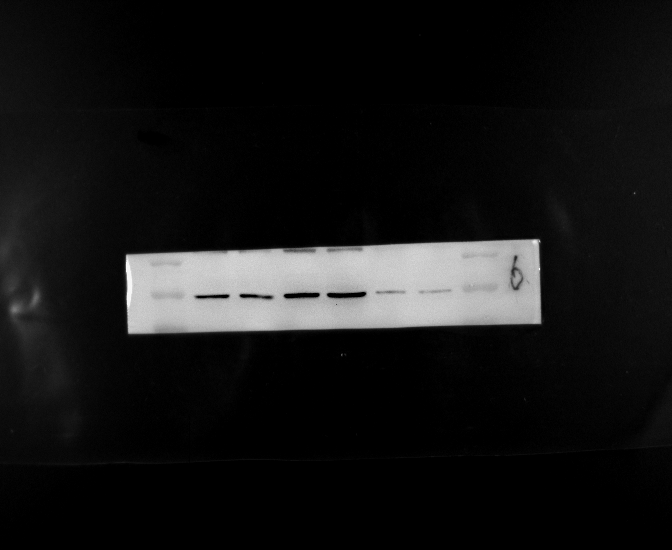

Supplement: Supplementary file 1 [file Data_Sheet_1.ZIP › 20210704-省三院-张琦-体内-全/GRP75(6)0707.tif]

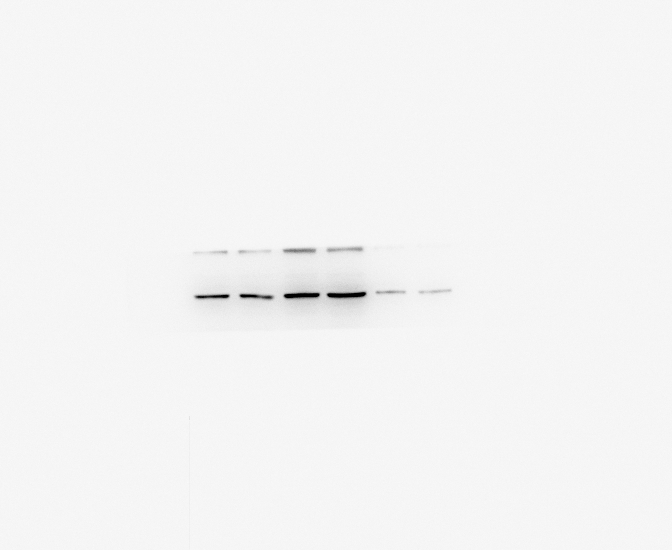

Supplement: Supplementary file 1 [file Data_Sheet_1.ZIP › 20210704-省三院-张琦-体内-全/GRP75(6)0707_chemi.tif]

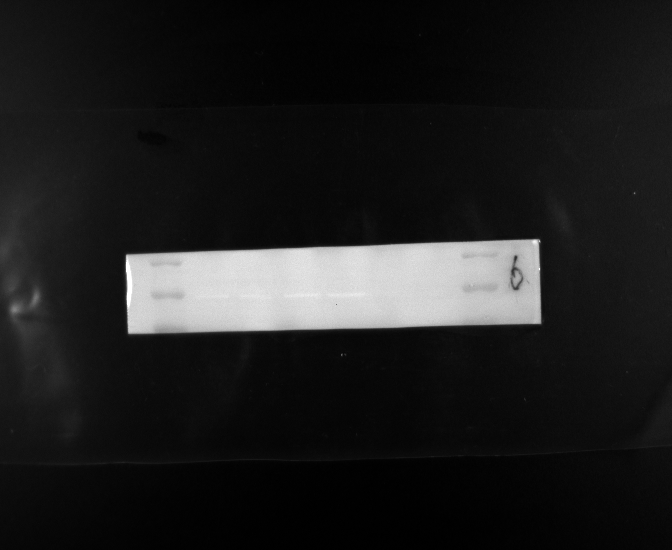

Supplement: Supplementary file 1 [file Data_Sheet_1.ZIP › 20210704-省三院-张琦-体内-全/GRP75(6)0707_marker.tif]

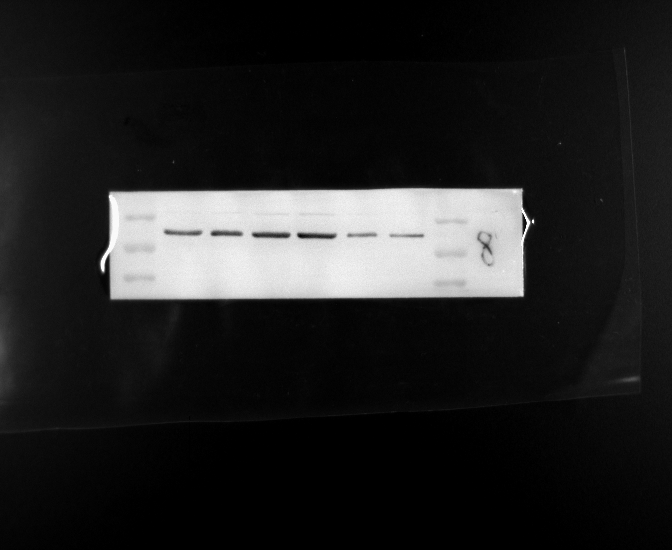

Supplement: Supplementary file 1 [file Data_Sheet_1.ZIP › 20210704-省三院-张琦-体内-全/GRP78(8)0707.tif]

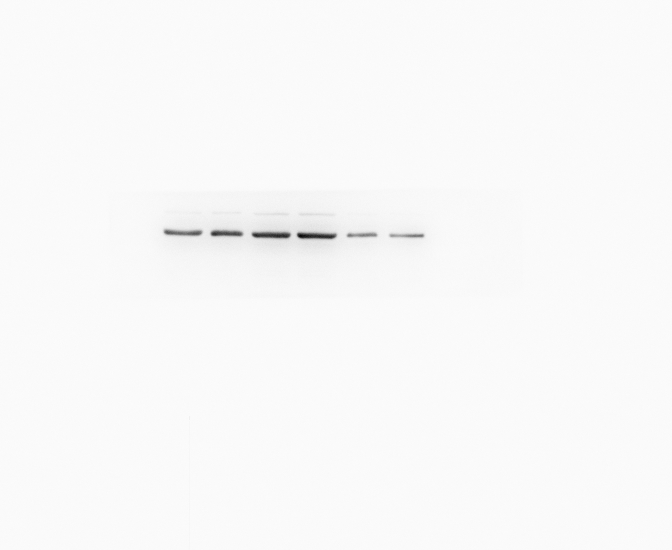

Supplement: Supplementary file 1 [file Data_Sheet_1.ZIP › 20210704-省三院-张琦-体内-全/GRP78(8)0707_chemi.tif]

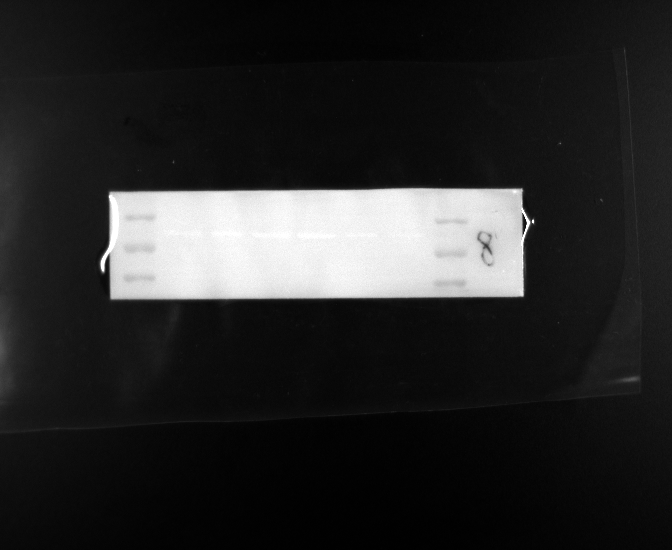

Supplement: Supplementary file 1 [file Data_Sheet_1.ZIP › 20210704-省三院-张琦-体内-全/GRP78(8)0707_marker.tif]

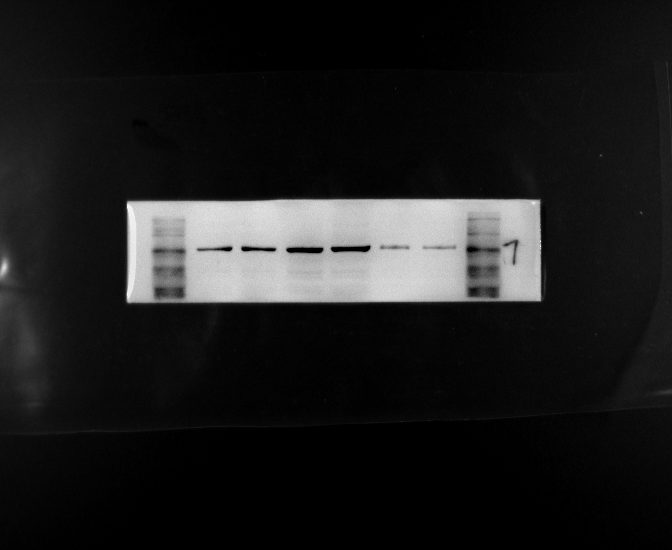

Supplement: Supplementary file 1 [file Data_Sheet_1.ZIP › 20210704-省三院-张琦-体内-全/GRP94(7)0707.tif]

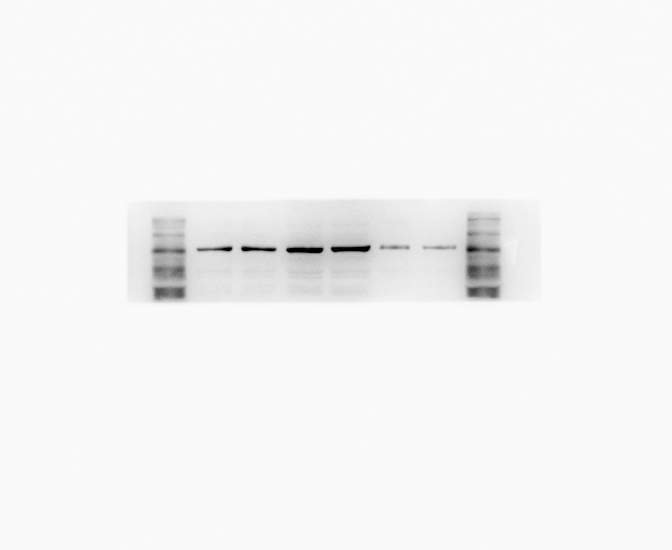

Supplement: Supplementary file 1 [file Data_Sheet_1.ZIP › 20210704-省三院-张琦-体内-全/GRP94(7)0707_chemi.tif]

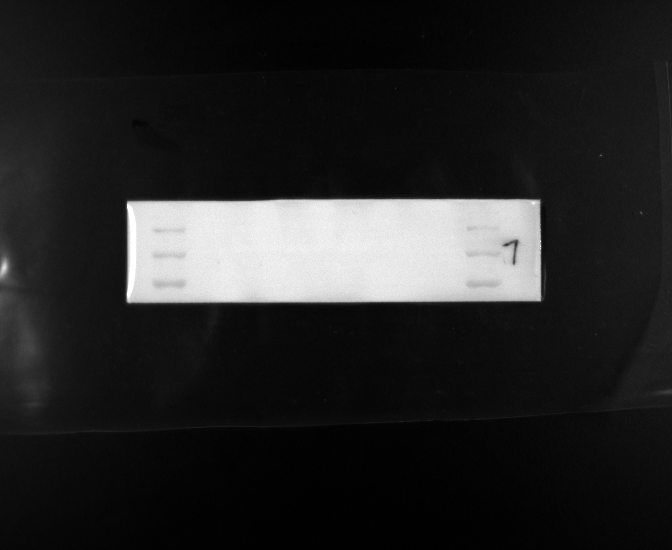

Supplement: Supplementary file 1 [file Data_Sheet_1.ZIP › 20210704-省三院-张琦-体内-全/GRP94(7)0707_marker.tif]

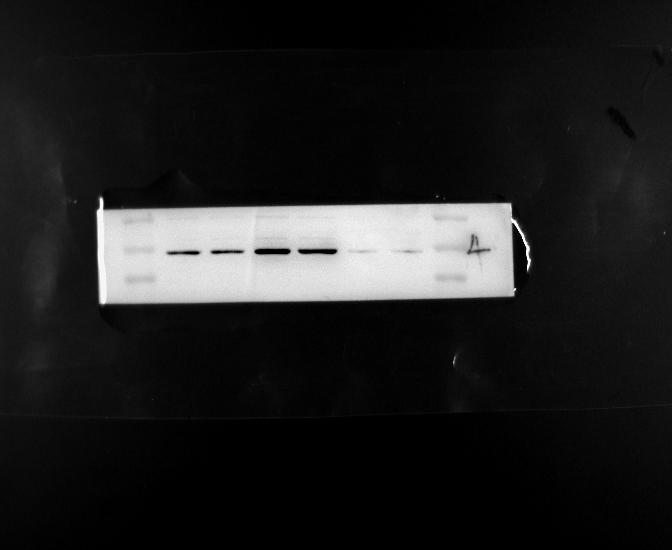

Supplement: Supplementary file 1 [file Data_Sheet_1.ZIP › 20210704-省三院-张琦-体内-全/MFN1(4)0707.tif]

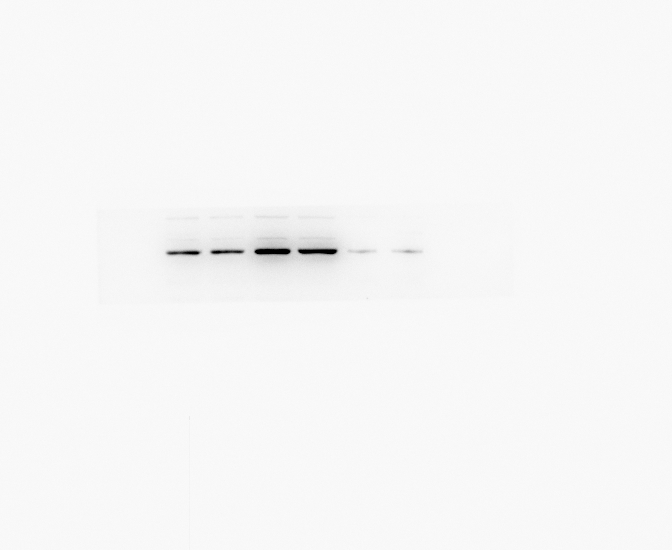

Supplement: Supplementary file 1 [file Data_Sheet_1.ZIP › 20210704-省三院-张琦-体内-全/MFN1(4)0707_chemi.tif]

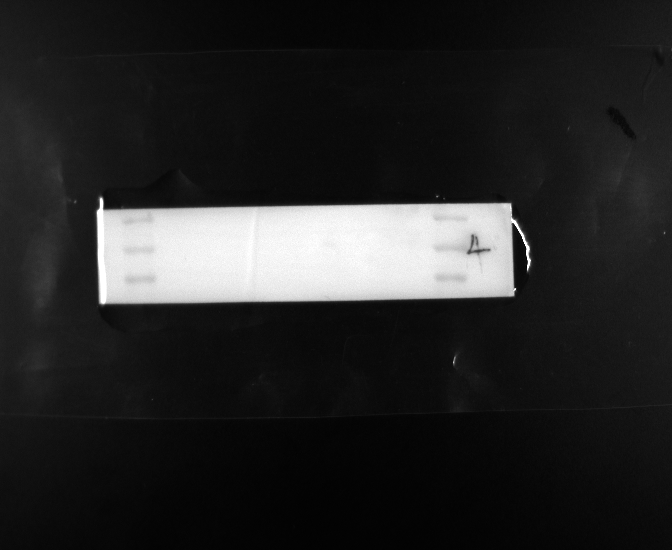

Supplement: Supplementary file 1 [file Data_Sheet_1.ZIP › 20210704-省三院-张琦-体内-全/MFN1(4)0707_marker.tif]

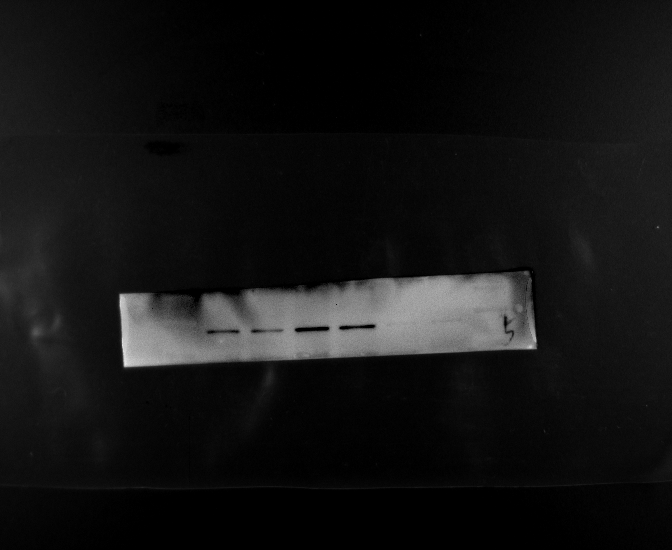

Supplement: Supplementary file 1 [file Data_Sheet_1.ZIP › 20210704-省三院-张琦-体内-全/MFN2(5)0707.tif]

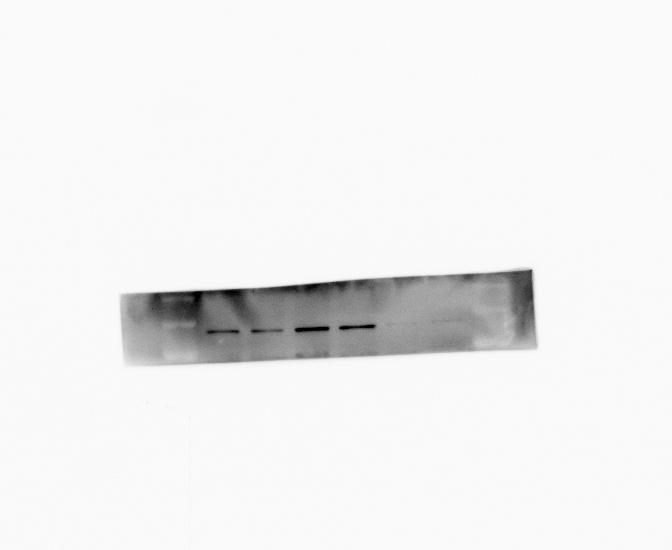

Supplement: Supplementary file 1 [file Data_Sheet_1.ZIP › 20210704-省三院-张琦-体内-全/MFN2(5)0707_chemi.tif]

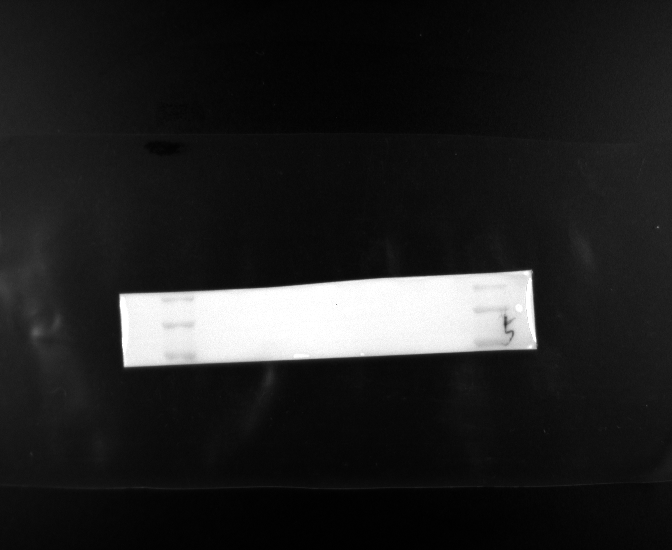

Supplement: Supplementary file 1 [file Data_Sheet_1.ZIP › 20210704-省三院-张琦-体内-全/MFN2(5)0707_marker.tif]

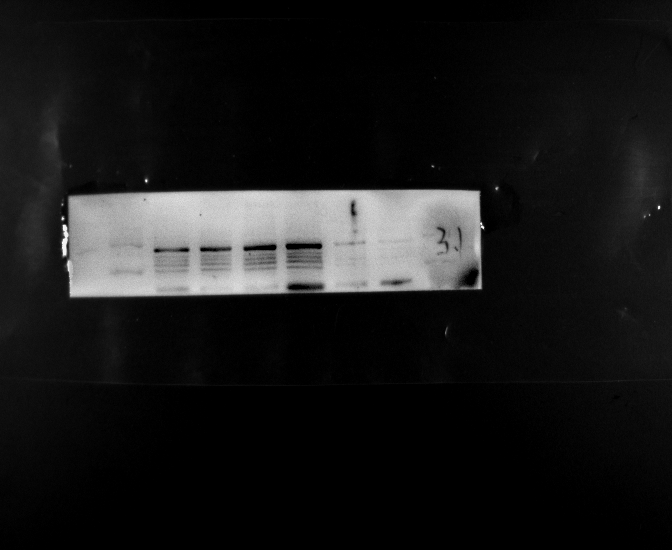

Supplement: Supplementary file 1 [file Data_Sheet_1.ZIP › 20210704-省三院-张琦-体内-全/P-IP3R(3-1)0707.tif]

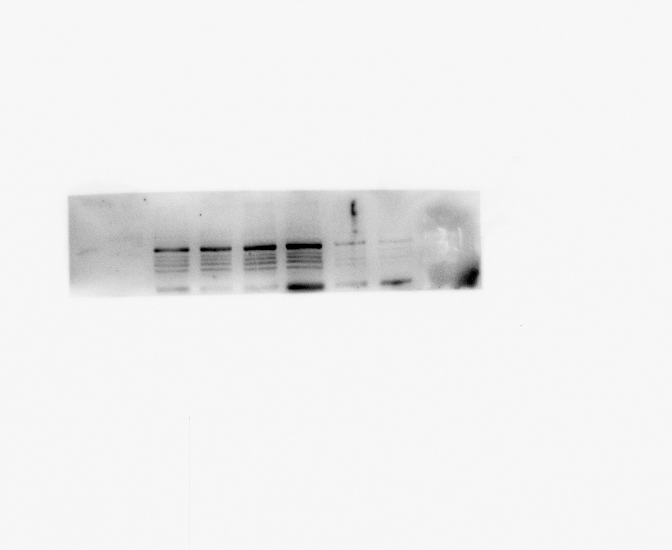

Supplement: Supplementary file 1 [file Data_Sheet_1.ZIP › 20210704-省三院-张琦-体内-全/P-IP3R(3-1)0707_chemi.tif]

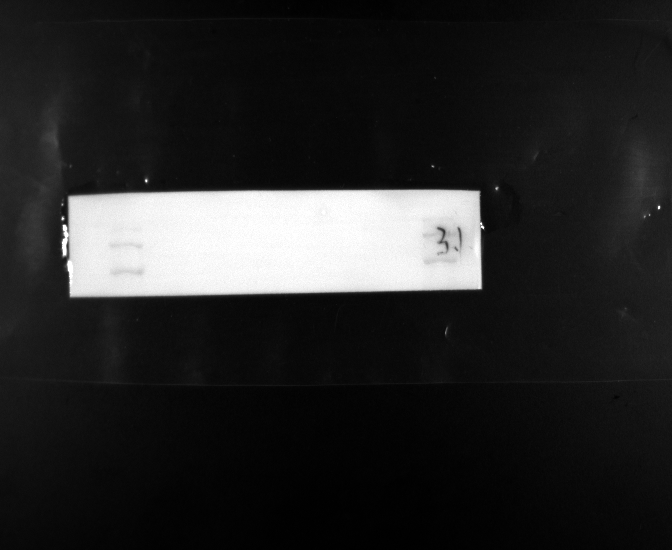

Supplement: Supplementary file 1 [file Data_Sheet_1.ZIP › 20210704-省三院-张琦-体内-全/P-IP3R(3-1)0707_marker.tif]

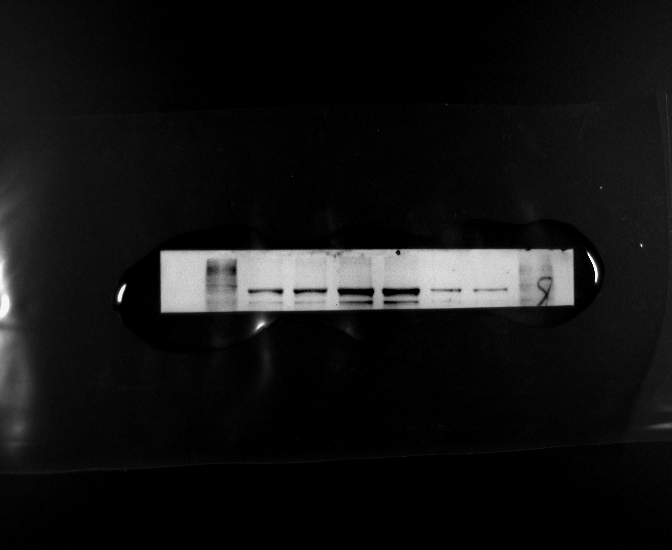

Supplement: Supplementary file 1 [file Data_Sheet_1.ZIP › 20210704-省三院-张琦-体内-全/P-PERK(9)0707-1.tif]

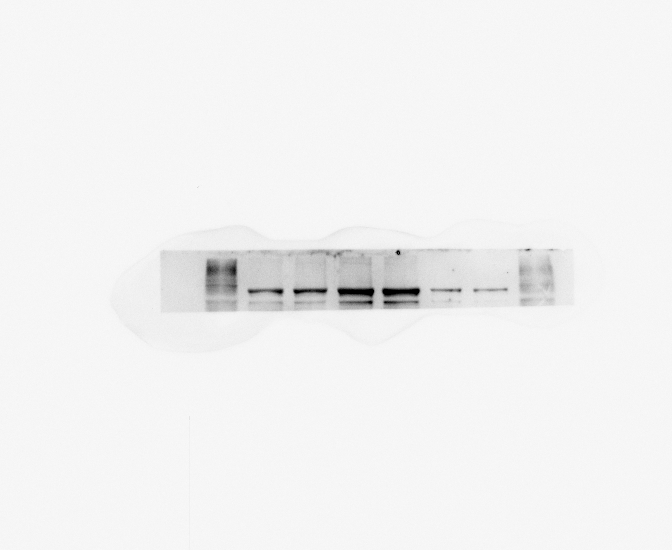

Supplement: Supplementary file 1 [file Data_Sheet_1.ZIP › 20210704-省三院-张琦-体内-全/P-PERK(9)0707-1_chemi.tif]

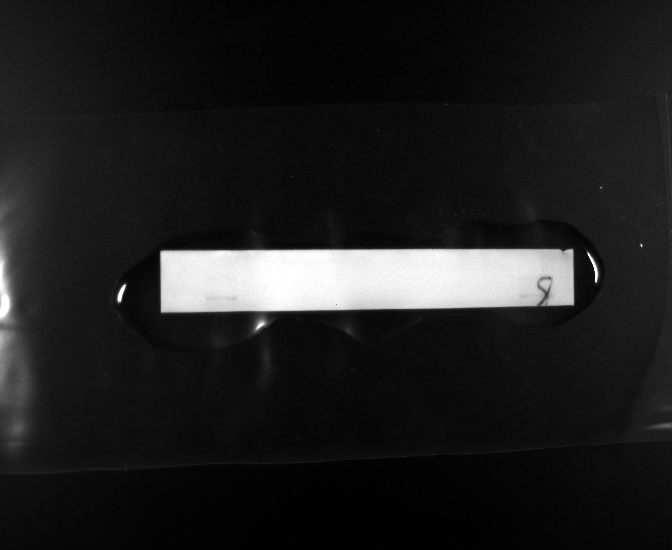

Supplement: Supplementary file 1 [file Data_Sheet_1.ZIP › 20210704-省三院-张琦-体内-全/P-PERK(9)0707-1_marker.tif]

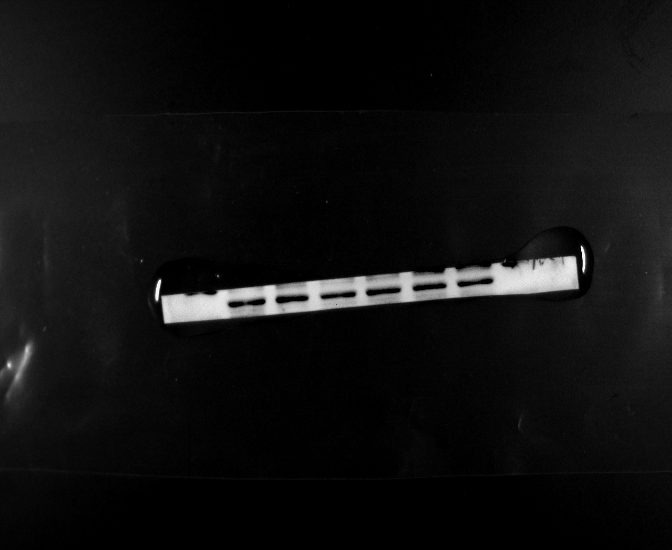

Supplement: Supplementary file 1 [file Data_Sheet_1.ZIP › 20210704-省三院-张琦-体内-全/t-IP3R(10-1)0707-2.tif]

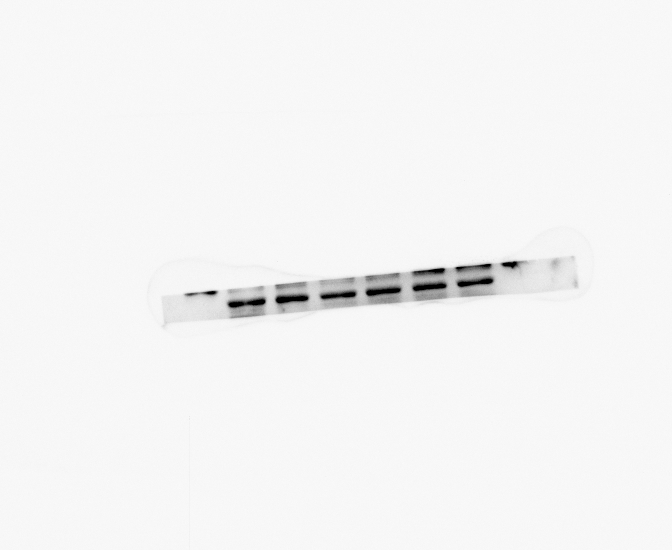

Supplement: Supplementary file 1 [file Data_Sheet_1.ZIP › 20210704-省三院-张琦-体内-全/t-IP3R(10-1)0707-2_chemi.tif]

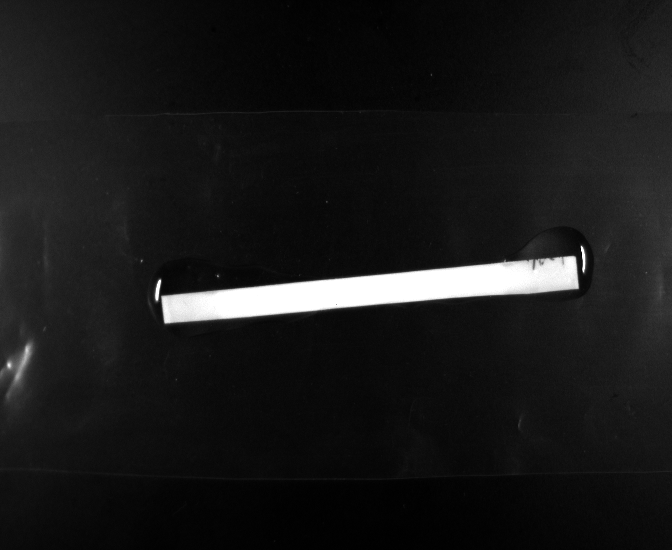

Supplement: Supplementary file 1 [file Data_Sheet_1.ZIP › 20210704-省三院-张琦-体内-全/t-IP3R(10-1)0707-2_marker.tif]

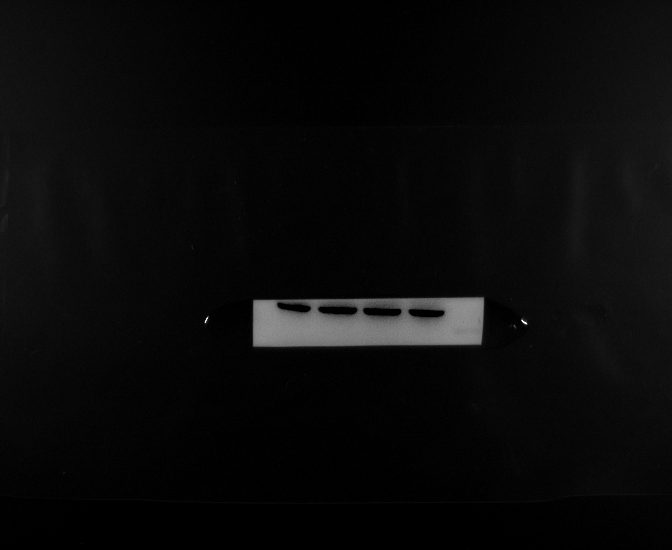

Supplement: Supplementary file 2 [file Data_Sheet_2.ZIP › 20210704-省三院-张琦-体外-全/actin(15-2-1)0621.tif]

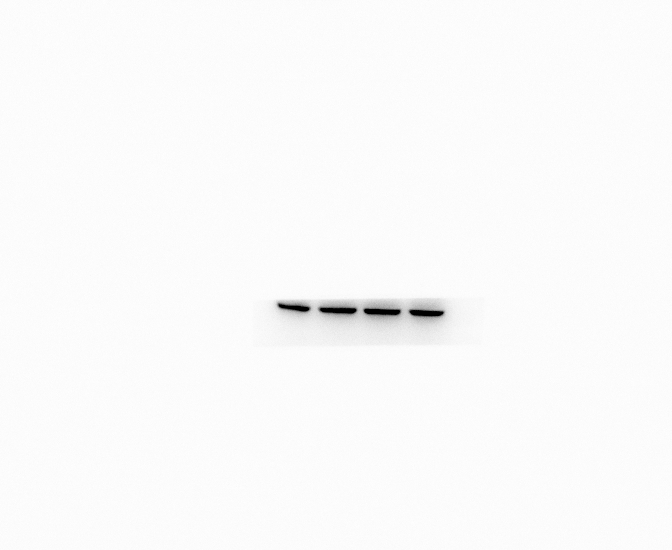

Supplement: Supplementary file 2 [file Data_Sheet_2.ZIP › 20210704-省三院-张琦-体外-全/actin(15-2-1)0621_chemi.tif]

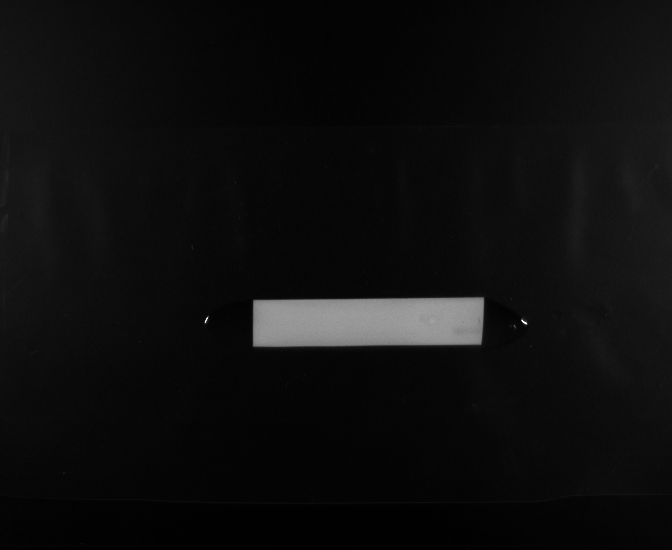

Supplement: Supplementary file 2 [file Data_Sheet_2.ZIP › 20210704-省三院-张琦-体外-全/actin(15-2-1)0621_marker.tif]

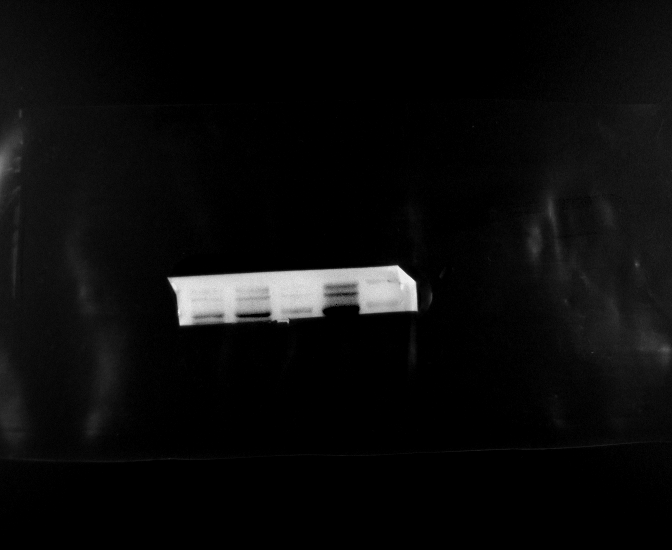

Supplement: Supplementary file 2 [file Data_Sheet_2.ZIP › 20210704-省三院-张琦-体外-全/ATF4(11-2-2)0707-1.tif]

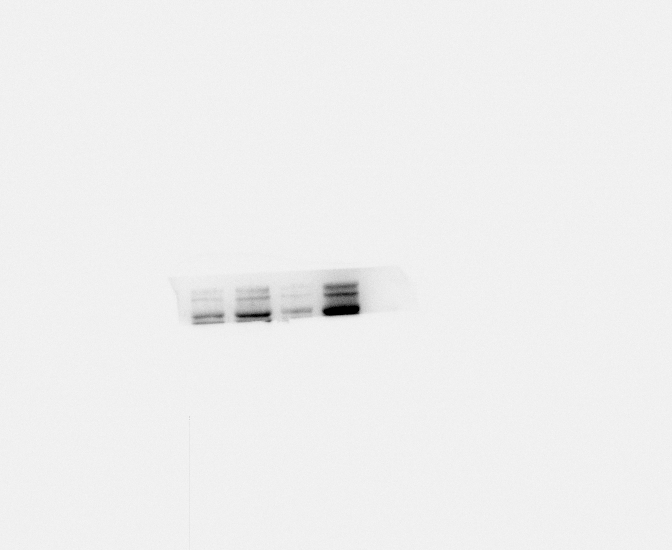

Supplement: Supplementary file 2 [file Data_Sheet_2.ZIP › 20210704-省三院-张琦-体外-全/ATF4(11-2-2)0707-1_chemi.tif]

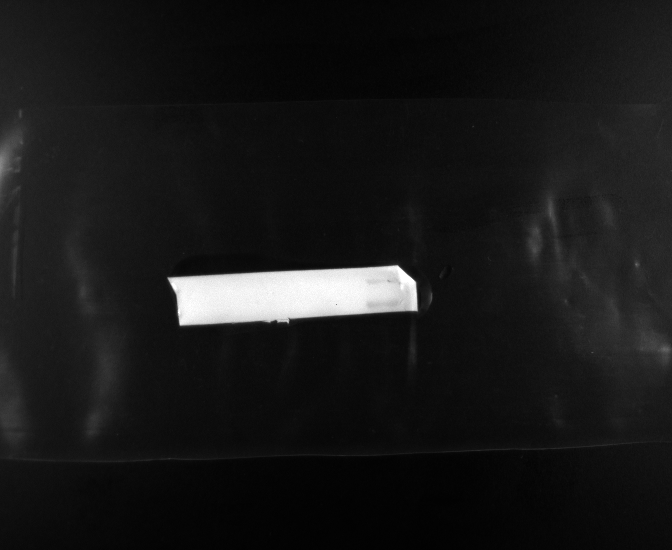

Supplement: Supplementary file 2 [file Data_Sheet_2.ZIP › 20210704-省三院-张琦-体外-全/ATF4(11-2-2)0707-1_marker.tif]

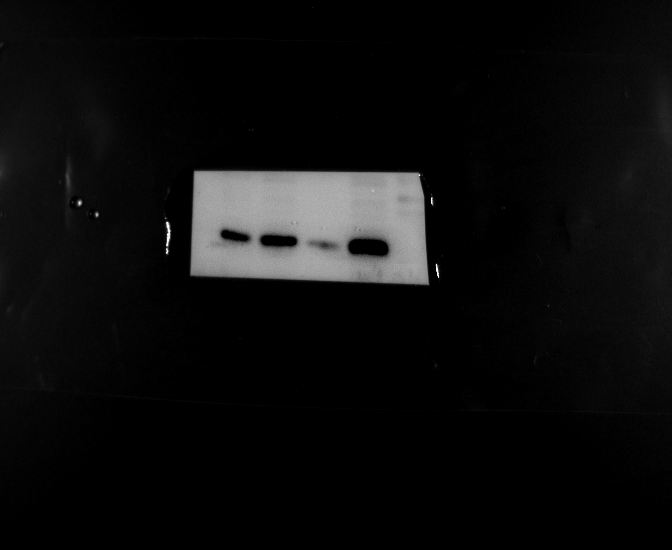

Supplement: Supplementary file 2 [file Data_Sheet_2.ZIP › 20210704-省三院-张琦-体外-全/BAX(14-2-1)0705.tif]

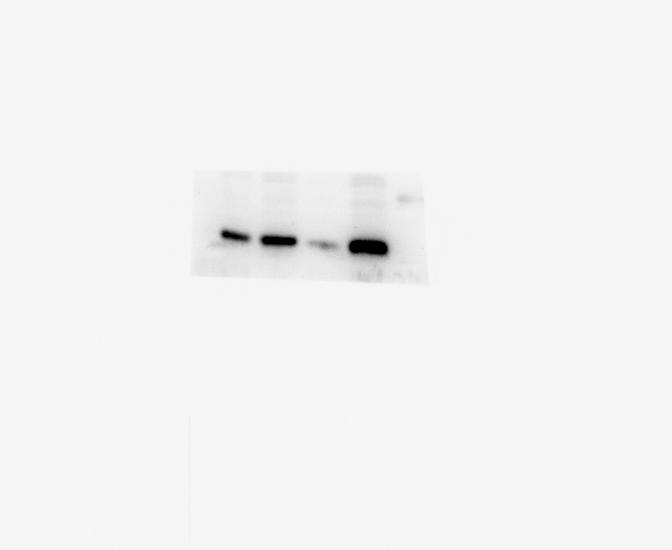

Supplement: Supplementary file 2 [file Data_Sheet_2.ZIP › 20210704-省三院-张琦-体外-全/BAX(14-2-1)0705_chemi.tif]

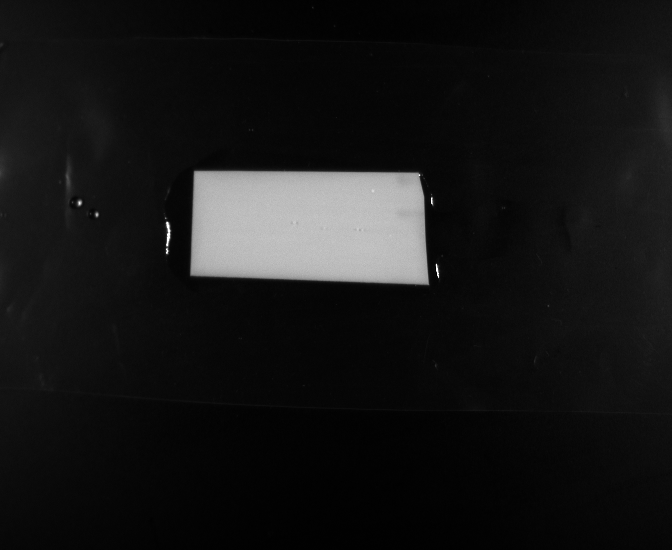

Supplement: Supplementary file 2 [file Data_Sheet_2.ZIP › 20210704-省三院-张琦-体外-全/BAX(14-2-1)0705_marker.tif]

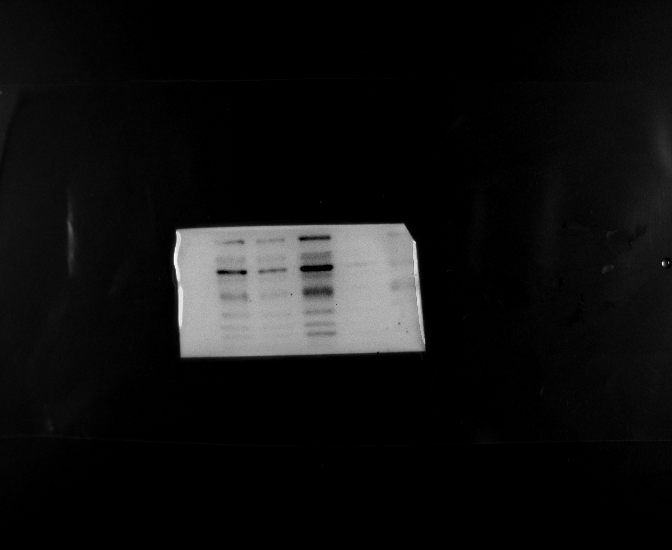

Supplement: Supplementary file 2 [file Data_Sheet_2.ZIP › 20210704-省三院-张琦-体外-全/BCL-2(15-1)0705.tif]

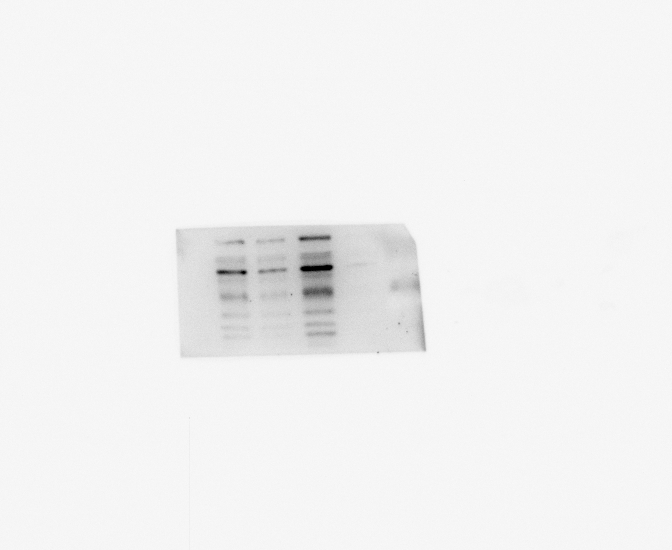

Supplement: Supplementary file 2 [file Data_Sheet_2.ZIP › 20210704-省三院-张琦-体外-全/BCL-2(15-1)0705_chemi.tif]

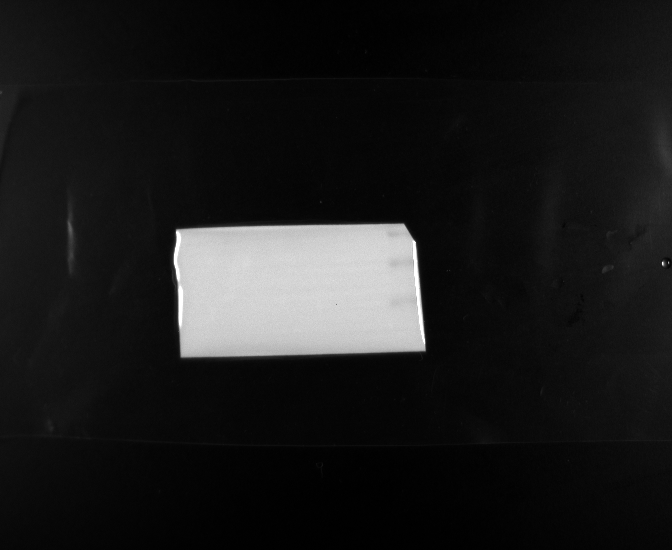

Supplement: Supplementary file 2 [file Data_Sheet_2.ZIP › 20210704-省三院-张琦-体外-全/BCL-2(15-1)0705_marker.tif]

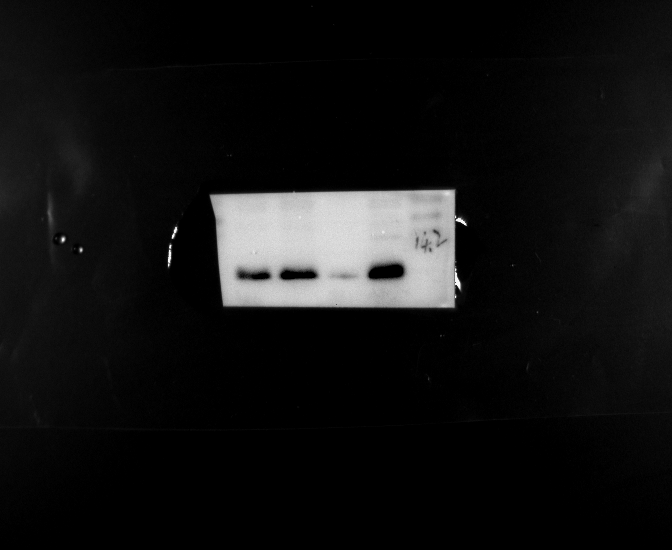

Supplement: Supplementary file 2 [file Data_Sheet_2.ZIP › 20210704-省三院-张琦-体外-全/CHOP(14-2-2)0705.tif]

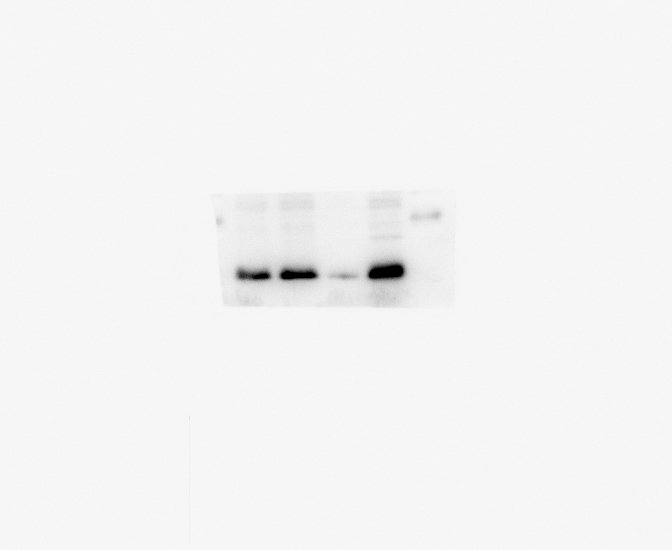

Supplement: Supplementary file 2 [file Data_Sheet_2.ZIP › 20210704-省三院-张琦-体外-全/CHOP(14-2-2)0705_chemi.tif]

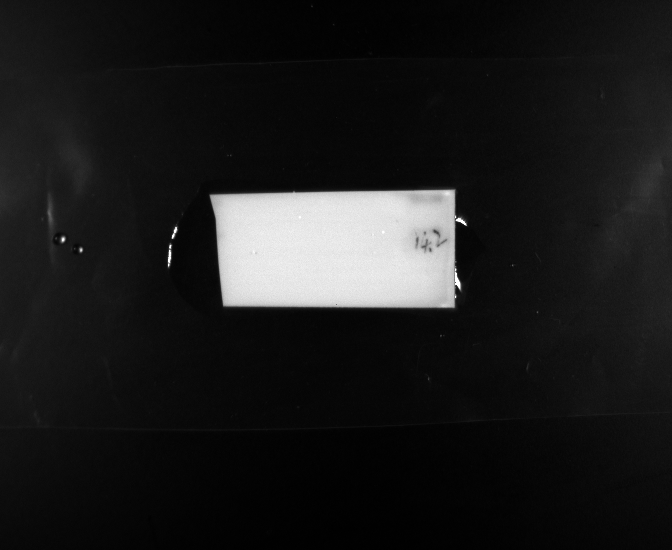

Supplement: Supplementary file 2 [file Data_Sheet_2.ZIP › 20210704-省三院-张琦-体外-全/CHOP(14-2-2)0705_marker.tif]

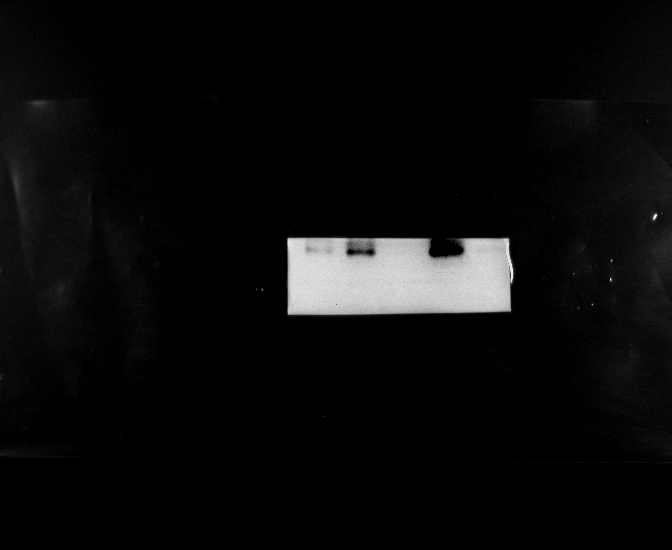

Supplement: Supplementary file 2 [file Data_Sheet_2.ZIP › 20210704-省三院-张琦-体外-全/cleaved caspase-3(13-2-1)0705-2.tif]

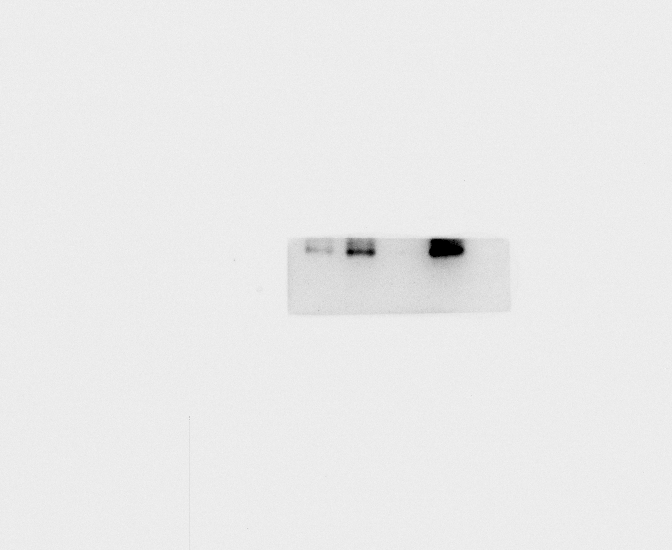

Supplement: Supplementary file 2 [file Data_Sheet_2.ZIP › 20210704-省三院-张琦-体外-全/cleaved caspase-3(13-2-1)0705-2_chemi.tif]

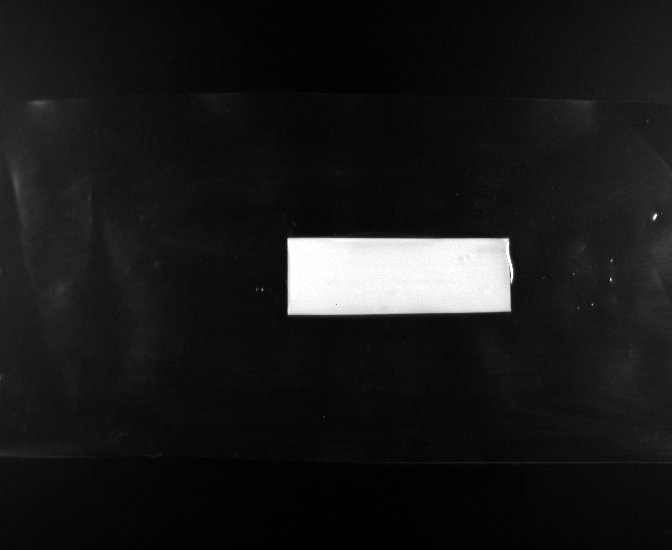

Supplement: Supplementary file 2 [file Data_Sheet_2.ZIP › 20210704-省三院-张琦-体外-全/cleaved caspase-3(13-2-1)0705-2_marker.tif]

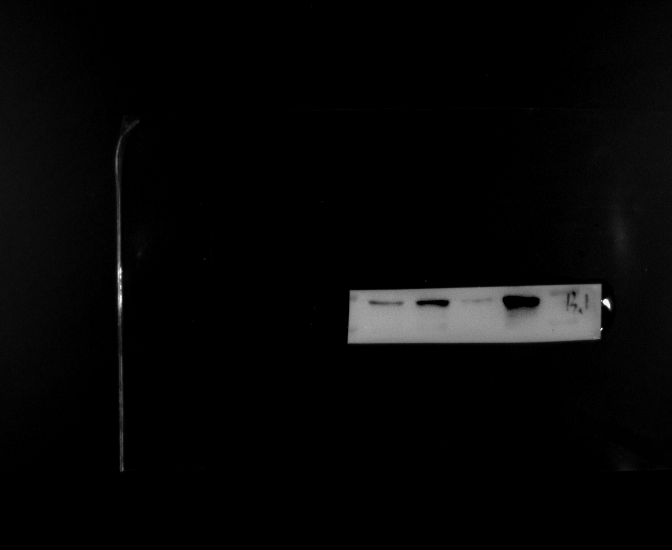

Supplement: Supplementary file 2 [file Data_Sheet_2.ZIP › 20210704-省三院-张琦-体外-全/cleaved caspase-9(13-1-2)0705-1.tif]

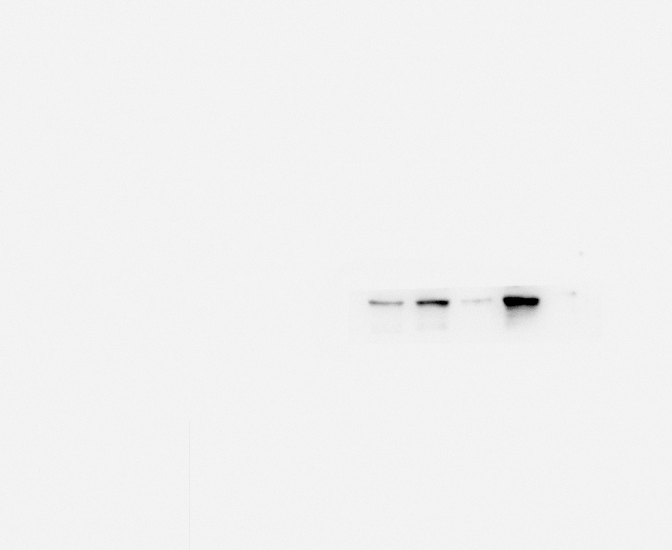

Supplement: Supplementary file 2 [file Data_Sheet_2.ZIP › 20210704-省三院-张琦-体外-全/cleaved caspase-9(13-1-2)0705-1_chemi.tif]

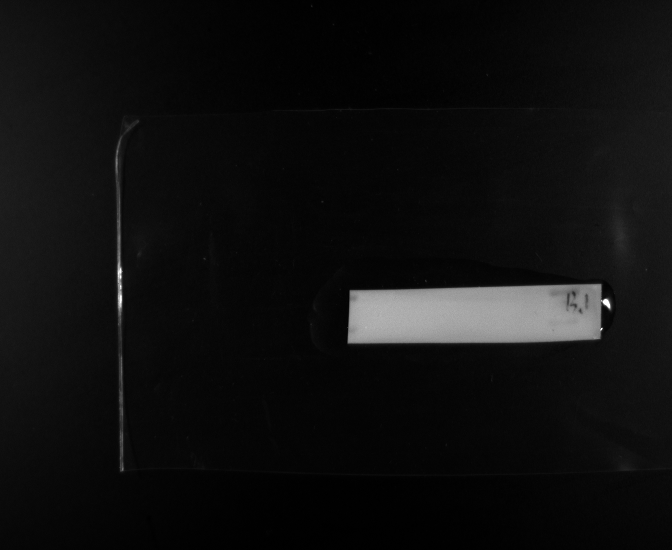

Supplement: Supplementary file 2 [file Data_Sheet_2.ZIP › 20210704-省三院-张琦-体外-全/cleaved caspase-9(13-1-2)0705-1_marker.tif]

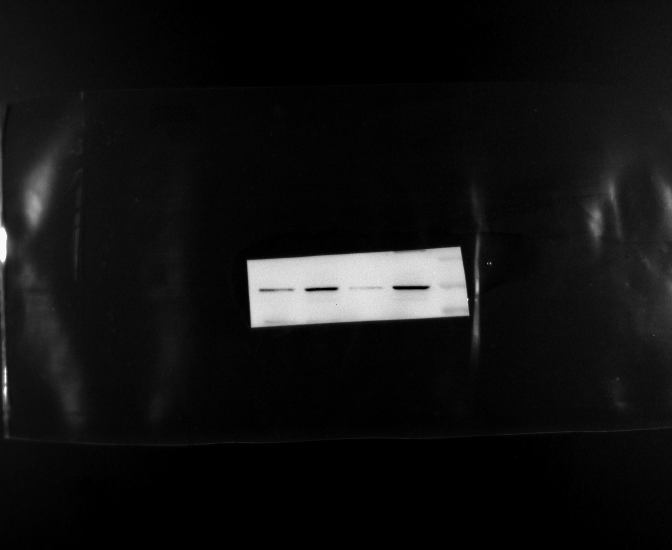

Supplement: Supplementary file 2 [file Data_Sheet_2.ZIP › 20210704-省三院-张琦-体外-全/GRP75(14-1)0707.tif]

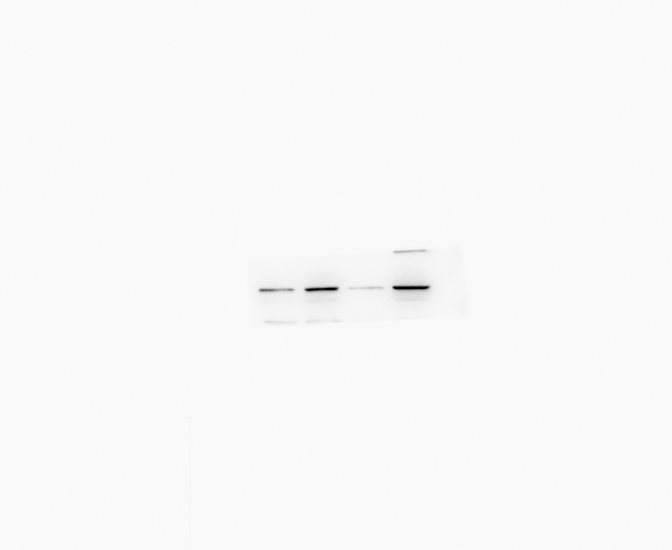

Supplement: Supplementary file 2 [file Data_Sheet_2.ZIP › 20210704-省三院-张琦-体外-全/GRP75(14-1)0707_chemi.tif]

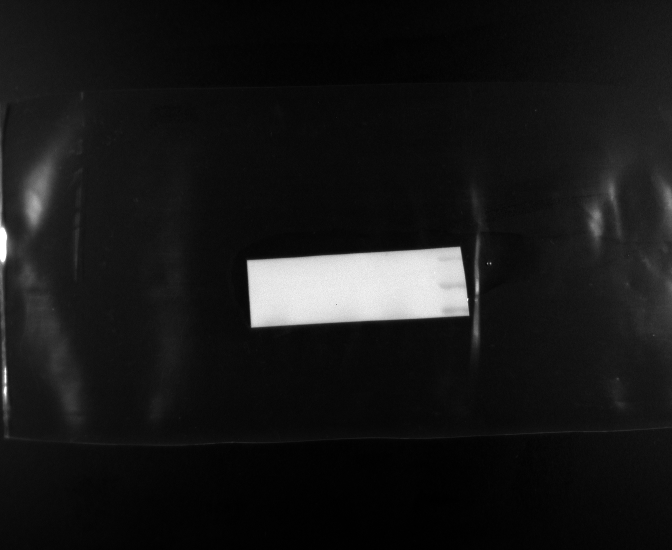

Supplement: Supplementary file 2 [file Data_Sheet_2.ZIP › 20210704-省三院-张琦-体外-全/GRP75(14-1)0707_marker.tif]

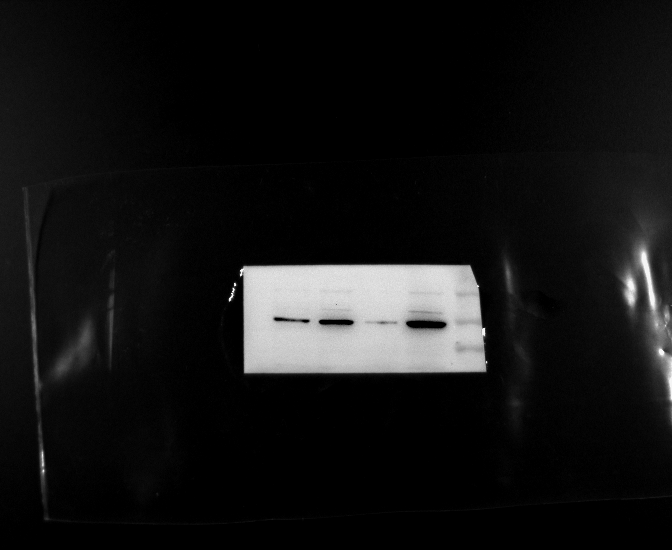

Supplement: Supplementary file 2 [file Data_Sheet_2.ZIP › 20210704-省三院-张琦-体外-全/MFN1(12-1)0707.tif]

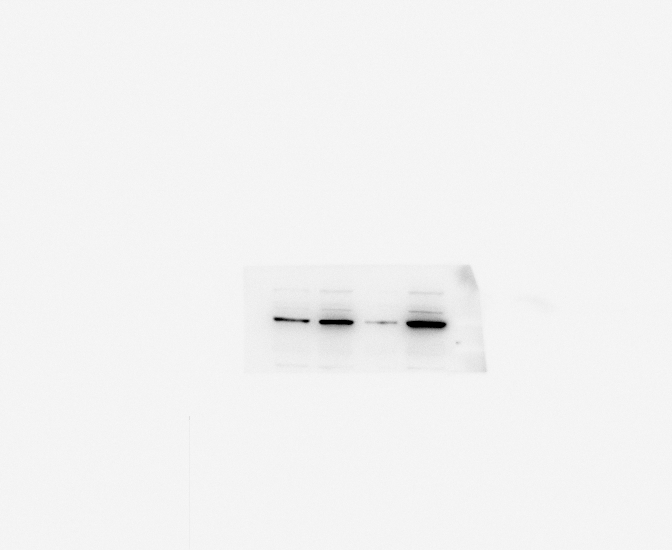

Supplement: Supplementary file 2 [file Data_Sheet_2.ZIP › 20210704-省三院-张琦-体外-全/MFN1(12-1)0707_chemi.tif]

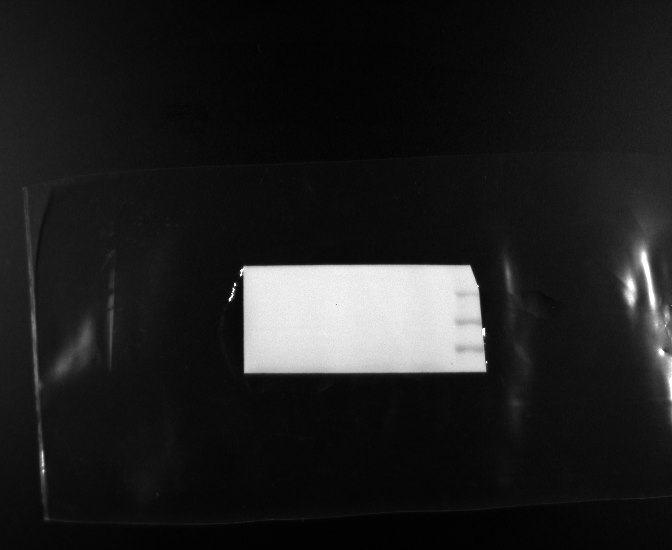

Supplement: Supplementary file 2 [file Data_Sheet_2.ZIP › 20210704-省三院-张琦-体外-全/MFN1(12-1)0707_marker.tif]

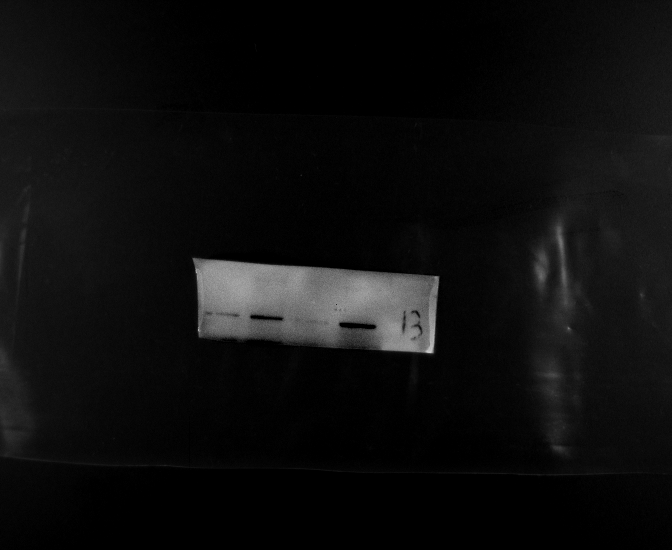

Supplement: Supplementary file 2 [file Data_Sheet_2.ZIP › 20210704-省三院-张琦-体外-全/MFN2(13-2)0707-1.tif]

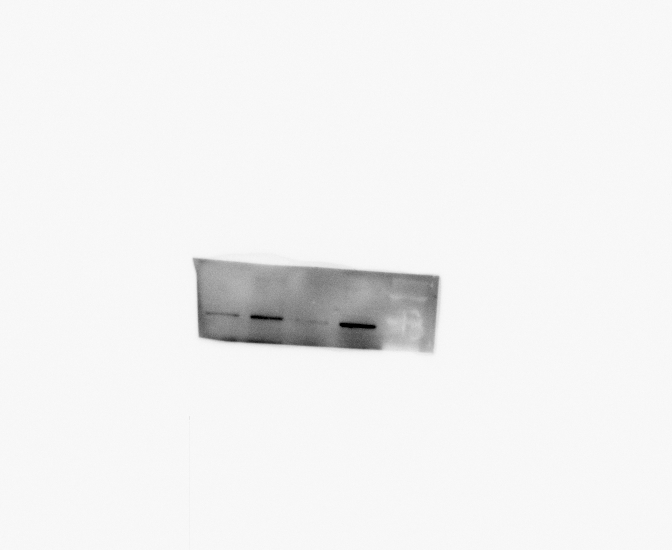

Supplement: Supplementary file 2 [file Data_Sheet_2.ZIP › 20210704-省三院-张琦-体外-全/MFN2(13-2)0707-1_chemi.tif]

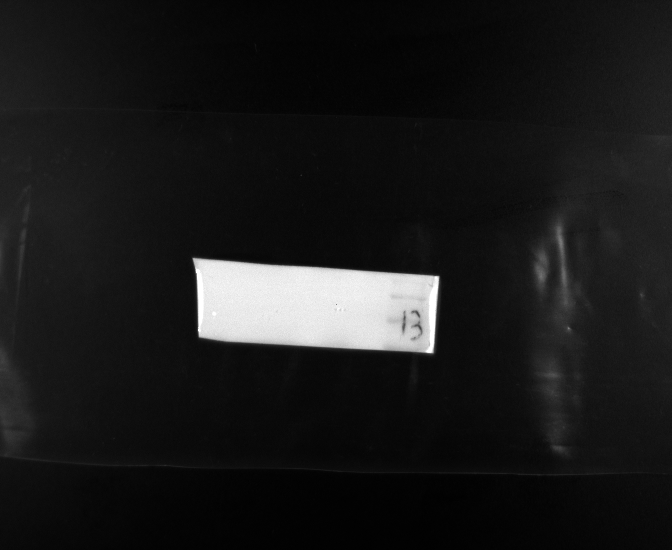

Supplement: Supplementary file 2 [file Data_Sheet_2.ZIP › 20210704-省三院-张琦-体外-全/MFN2(13-2)0707-1_marker.tif]

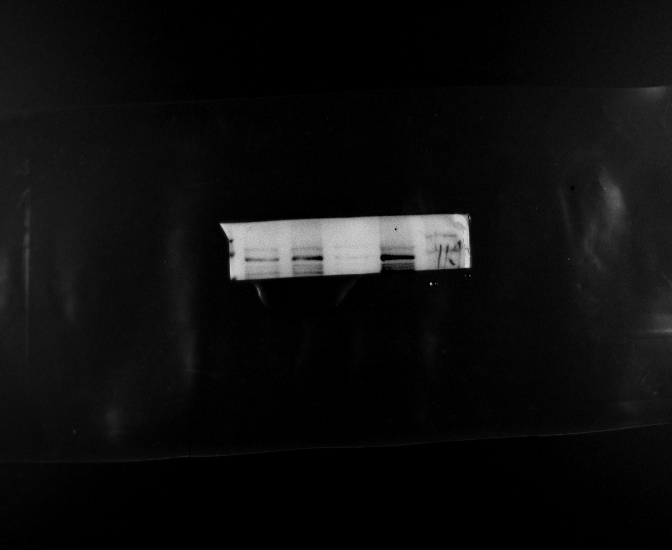

Supplement: Supplementary file 2 [file Data_Sheet_2.ZIP › 20210704-省三院-张琦-体外-全/P-IP3R(11-1-2)0707-2.tif]

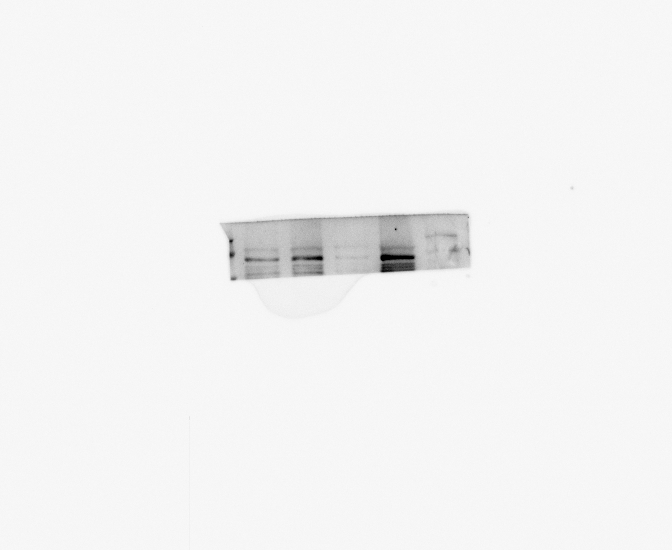

Supplement: Supplementary file 2 [file Data_Sheet_2.ZIP › 20210704-省三院-张琦-体外-全/P-IP3R(11-1-2)0707-2_chemi.tif]

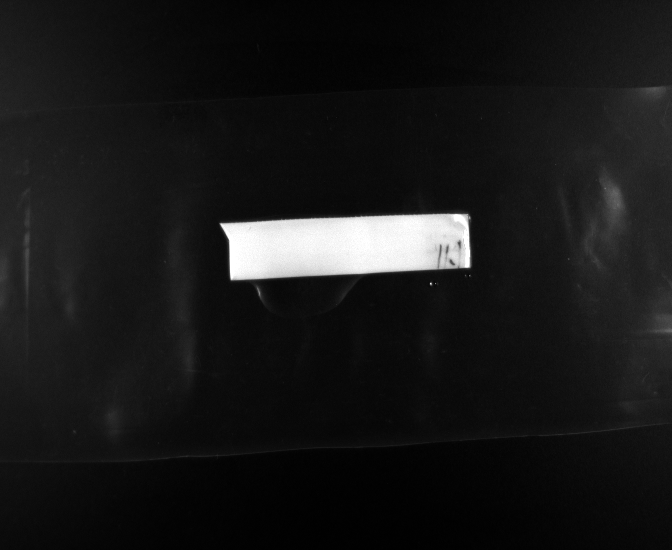

Supplement: Supplementary file 2 [file Data_Sheet_2.ZIP › 20210704-省三院-张琦-体外-全/P-IP3R(11-1-2)0707-2_marker.tif]

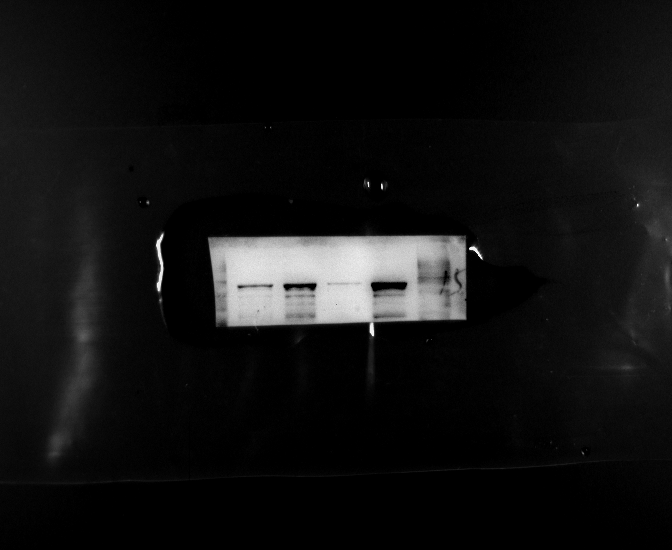

Supplement: Supplementary file 2 [file Data_Sheet_2.ZIP › 20210704-省三院-张琦-体外-全/P-PERK(15-2)0707.tif]

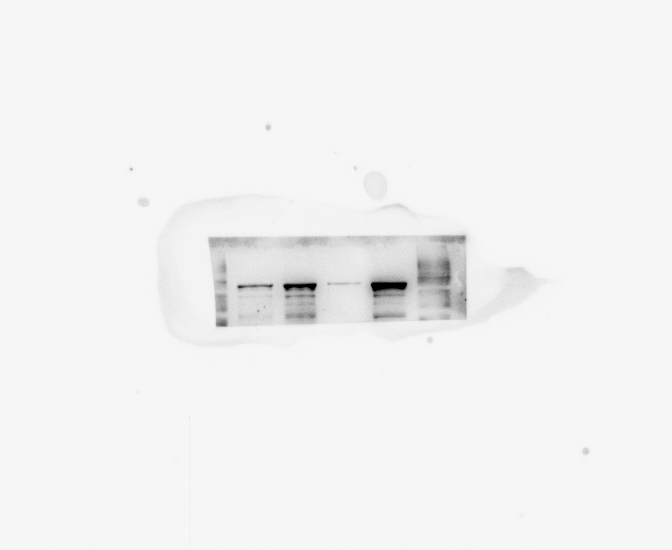

Supplement: Supplementary file 2 [file Data_Sheet_2.ZIP › 20210704-省三院-张琦-体外-全/P-PERK(15-2)0707_chemi.tif]

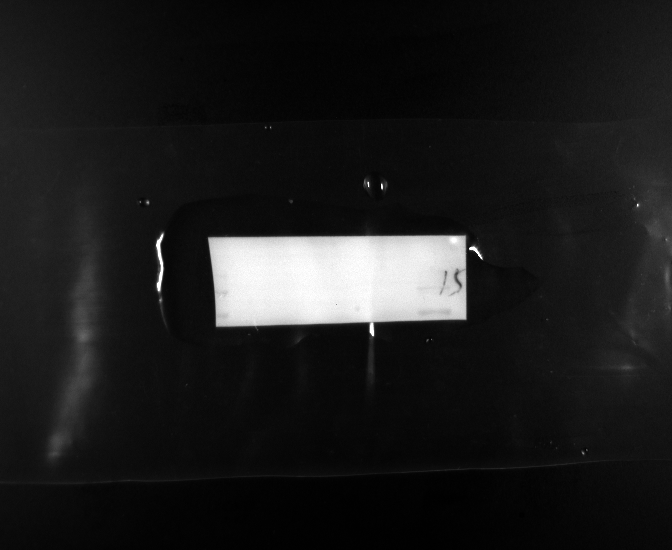

Supplement: Supplementary file 2 [file Data_Sheet_2.ZIP › 20210704-省三院-张琦-体外-全/P-PERK(15-2)0707_marker.tif]

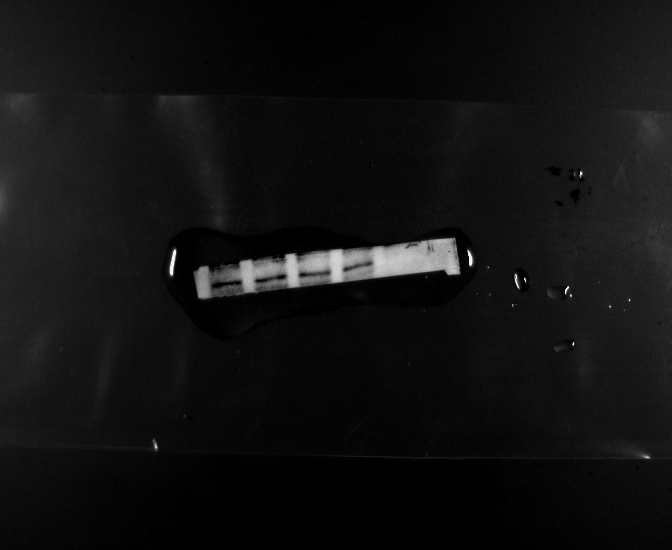

Supplement: Supplementary file 2 [file Data_Sheet_2.ZIP › 20210704-省三院-张琦-体外-全/t-IP3R(2-1-1)0709-4.tif]

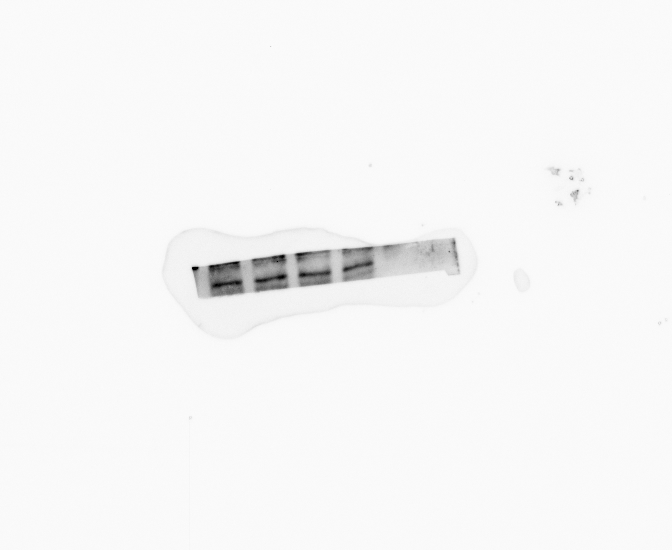

Supplement: Supplementary file 2 [file Data_Sheet_2.ZIP › 20210704-省三院-张琦-体外-全/t-IP3R(2-1-1)0709-4_chemi.tif]

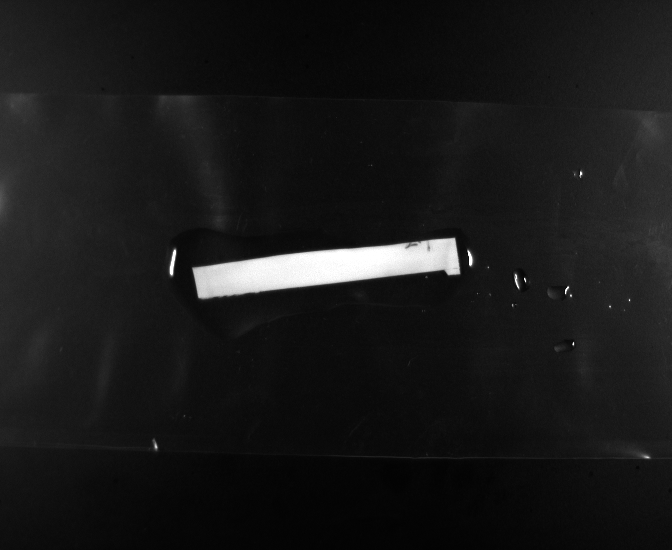

Supplement: Supplementary file 2 [file Data_Sheet_2.ZIP › 20210704-省三院-张琦-体外-全/t-IP3R(2-1-1)0709-4_marker.tif]

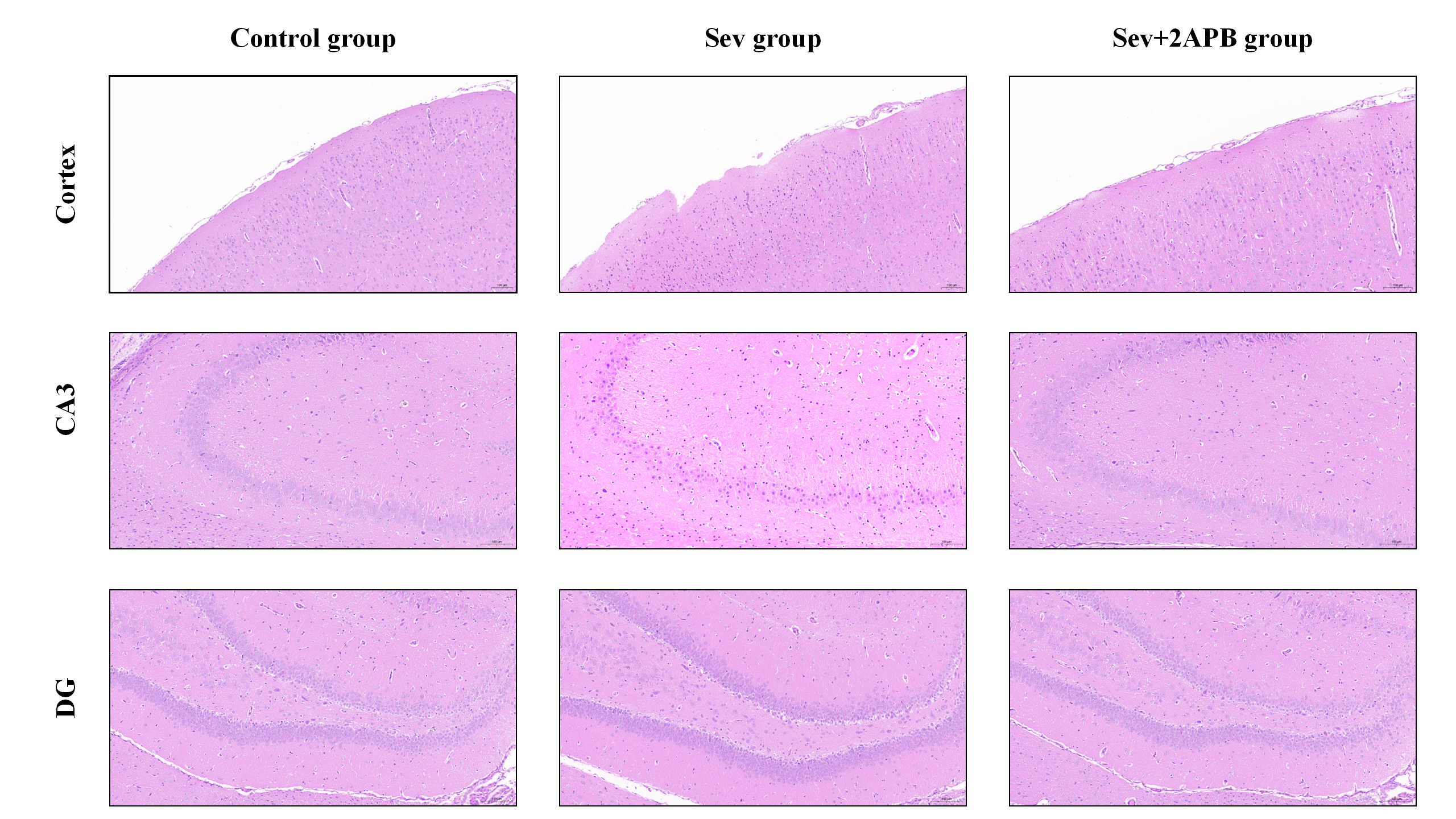

Supplement: Supplementary file 3 [file Image_1.TIF]

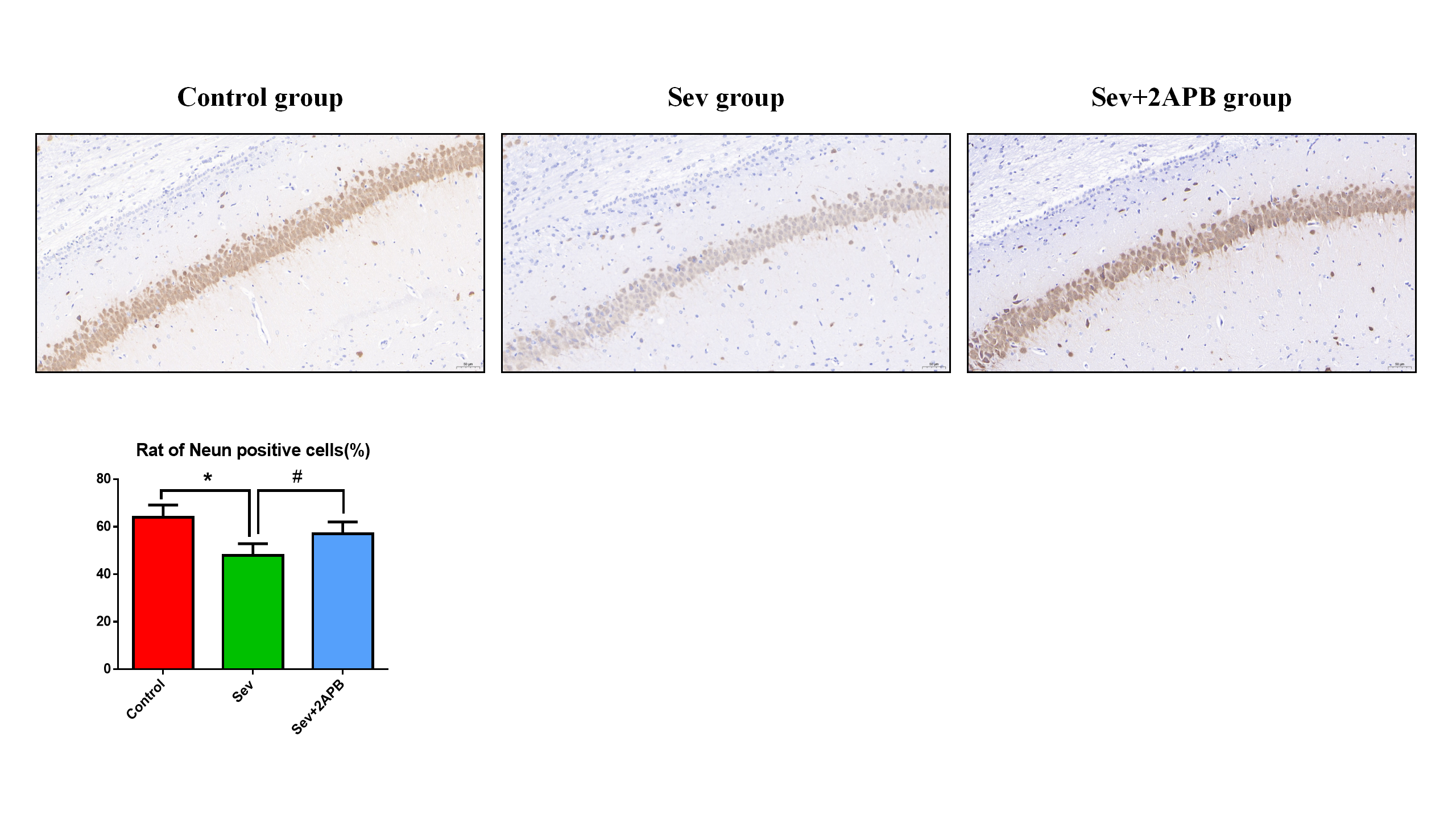

Supplement: Supplementary file 4 [file Image_2.TIF]

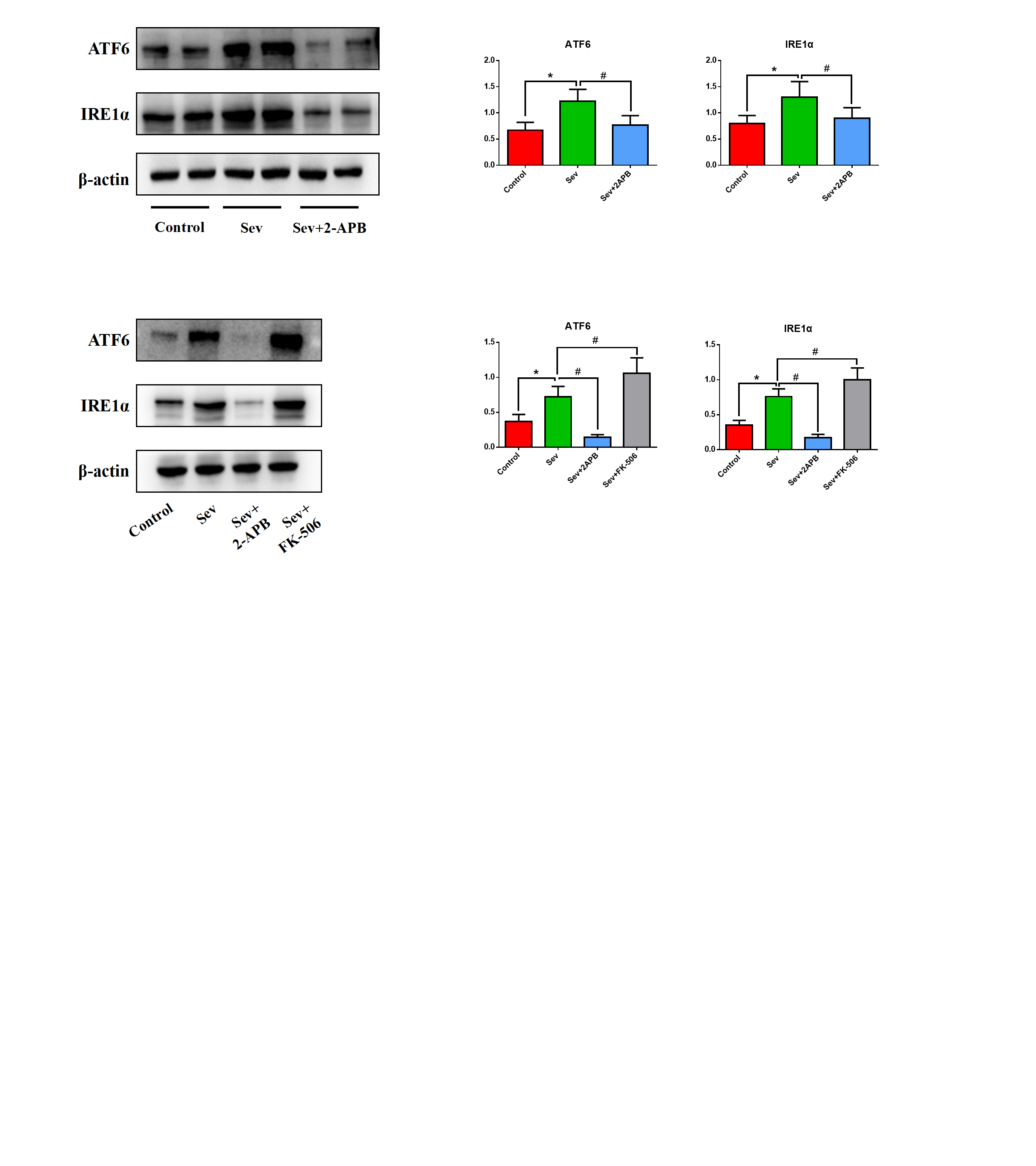

Supplement: Supplementary file 5 [file Image_3.TIF]

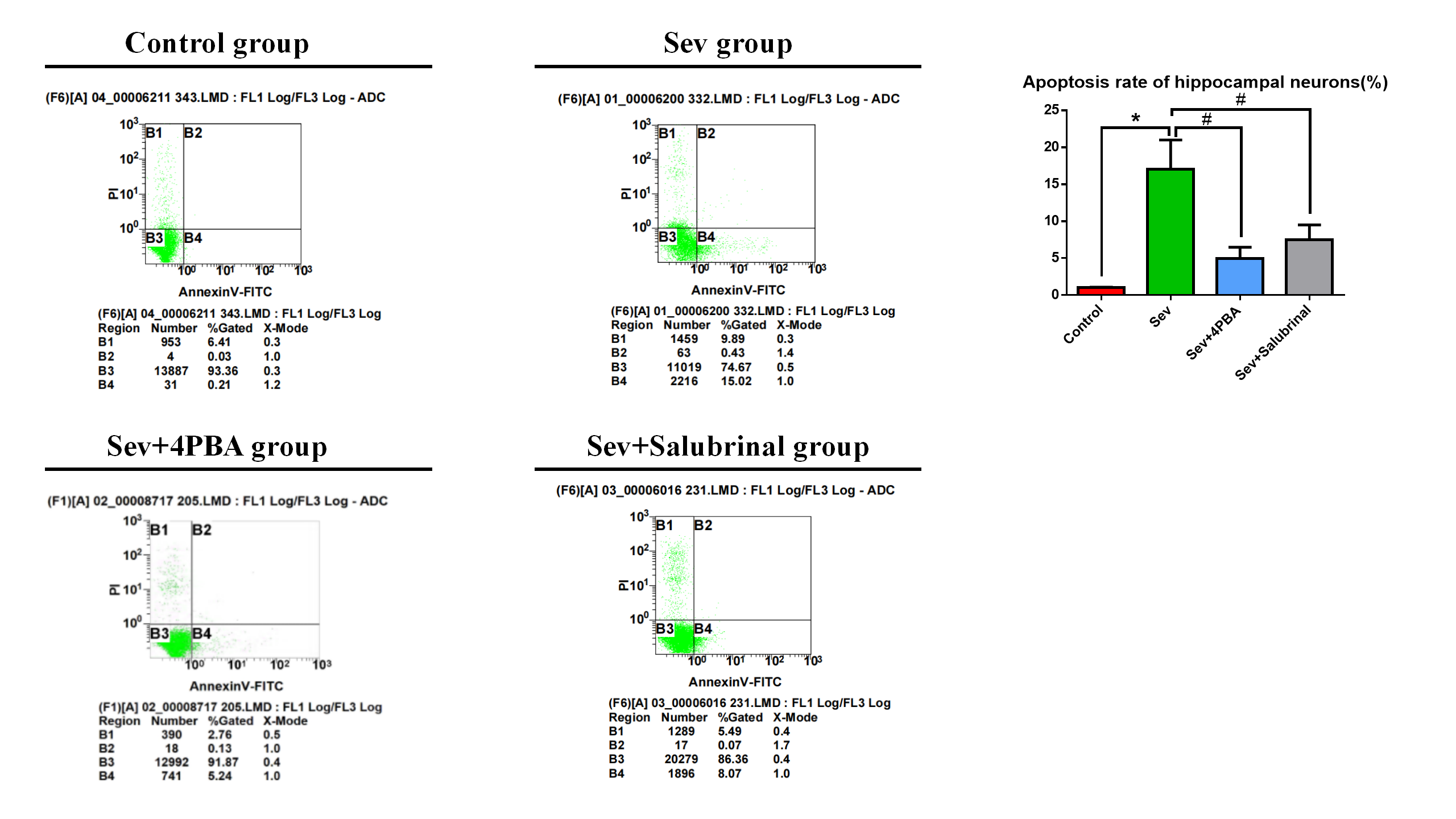

Supplement: Supplementary file 6 [file Image_4.TIF]

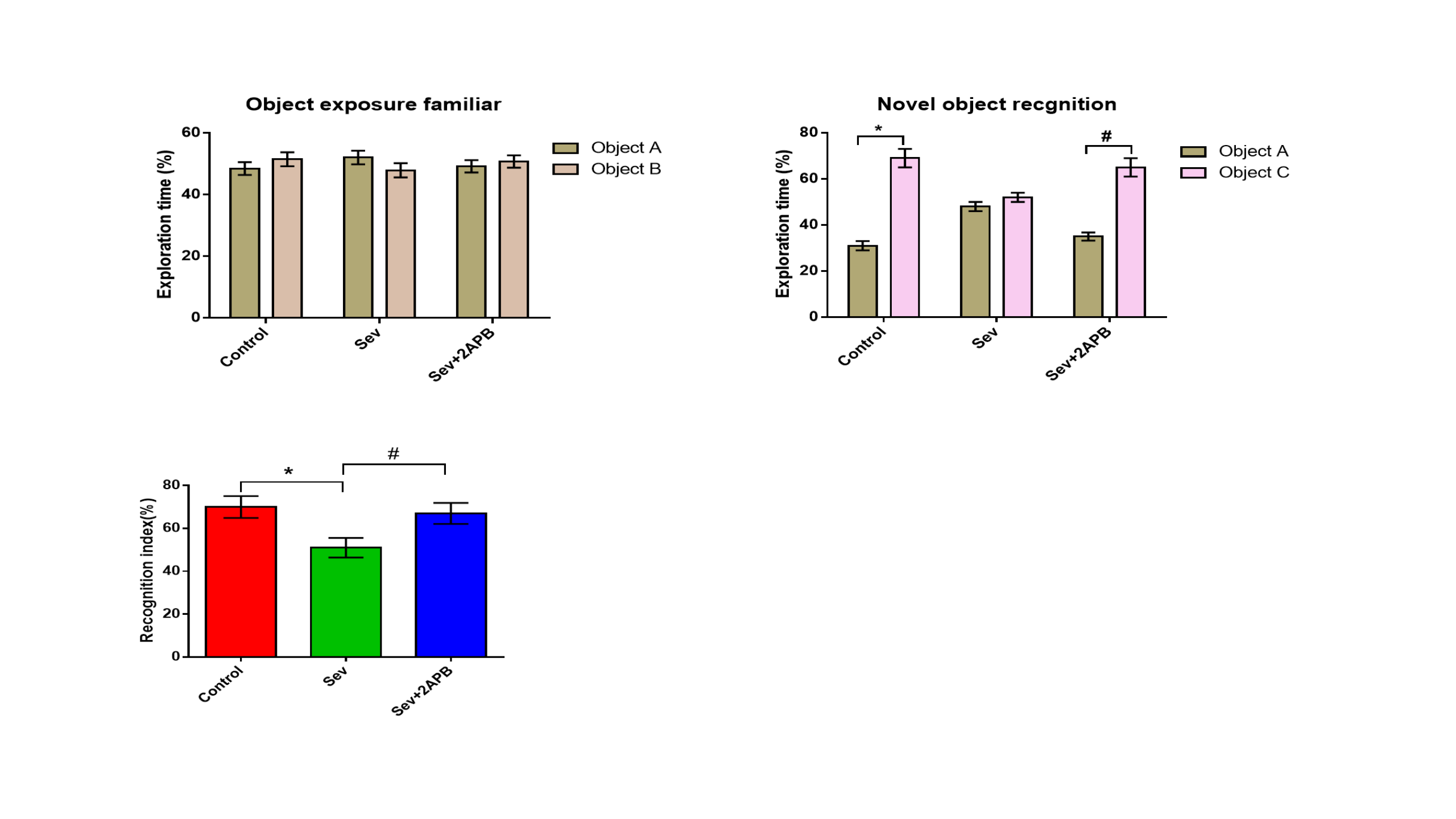

Supplement: Supplementary file 7 [file Image_5.TIF]
